# Digital science communication for sustainability literacy: A scoping review

**DOI:** 10.12688/openreseurope.20966.1

**Published:** 2025-08-08

**Authors:** Tin Shine Aung, Cláudia Faria, Joana Sousa, Mónica Mendes

**Affiliations:** 1 Ciências da Sustentabilidade, CIEBA, ITI-LARSyS, Universidade de Lisboa, Alameda da Universidade, 1649-004 Lisboa, Portugal; 2 UIDEF, Instituto de Educação, Universidade de Lisboa, Alameda da Universidade, 1649-013 Lisbon, Portugal; 3 Instituto de Saúde Ambiental, Faculdade de Medicina, Universidade de Lisboa, Av. Prof. Egas Moniz MB, 1649-028 Lisboa, Portugal; 4 ITI-LARSyS, Faculdade de Belas-Artes, Universidade de Lisboa, Largo da Academia Nacional de Belas-Artes, 1249-058 Lisboa, Portugal

**Keywords:** Behaviour Change, Digital Science Communication, Education for Sustainable Development (ESD), Information and Communication Technology (ICT), Sustainability Literacy

## Abstract

**Background:**

Global challenges such as climate change and resource depletion stem from unsustainable human activities, highlighting the critical need for widespread sustainability literacy to achieve the Sustainable Development Goals (SDGs). Education for Sustainable Development (ESD) is a key enabler, fostering holistic learning and action across all levels. Given this critical need, integrating digital science communication into ESD is essential.

**Objective:**

This scoping review explores how digital science communication contributes to sustainability literacy within ESD, focusing on advancing SDG targets 4.7, 12.8, and 13.3.

**Design:**

Guided by the PRISMA-ScR framework, we systematically reviewed 20 peer-reviewed articles published between 2000 and 2023. An initial search was conducted across Scopus and Web of Science, with the final eligibility screening and analysis based on the Web of Science dataset, selected for its robust filtering capabilities within educational and sustainability contexts.

**Results:**

Thematic synthesis using NVIVO 12 revealed that digital tools, such as XR technologies, serious games, educational videos, and online learning platforms, enhance sustainability literacy through interactive engagement, contextualized learning, and flexible access.

**Conclusion:**

However, challenges remain, including limited evidence of long-term behavioral impacts and the need for more tailored digital content. Ultimately, this review suggests that digital communication can help reconnect people with nature and deepen their understanding of sustainable practices, while highlighting the need for further research to address persistent gaps.

## Plain language summary for Open Research Europe (ORE)

Climate change, pollution, and the overuse of natural resources are major global problems caused by how people live and use the planet’s resources. People everywhere should understand how to live sustainably to solve these challenges and achieve the United Nations Sustainable Development Goals (SDGs). This understanding is called sustainability literacy, which means having the knowledge, skills, and values to make thoughtful and responsible decisions supporting a healthy planet, fair societies, and strong communities. One way to build sustainability literacy is through Education for Sustainable Development (ESD), which helps people learn how to contribute to a better future. Today, digital tools like virtual reality, edutainment games, videos, and online learning platforms are used in schools, universities, and communities to support this learning. This review examined 20 research articles published between 2000 and 2023 to determine how well these digital tools help people understand sustainability. Using a method called scoping review and a software program called NVIVO12, we found that digital tools can make learning more interactive, flexible, and connected to real-world issues. These tools help learners understand complex sustainability topics better and encourage them to think and act differently. However, the review also showed that we need more research to know if these tools lead to long-term behavior change and to improve how these tools are designed and used. In short, digital science communication can play an important role in helping people understand and adopt sustainable practices.

## 1. Introduction

The world faces many formidable challenges, including the interconnected crises of food, finance, climate, and global recession, which have significant implications for sustainable development and environmental management ( UNDESA, 2022). These ongoing climate change crises, far-reaching consequences, and the exploitation of natural resources are intricately linked to human behaviors. Humans' unsustainable actions have fundamentally altered the Earth's ecosystem, directly threatening human lives. The evidence of dramatic and rapid global warming is indisputable, with two-thirds of the 1°C increases in global temperatures occurring in just the past century, highlighting the human impact and responsibility ( UNESCO, 2020). According to the International Panel on Climate Change (IPCC), we must make rapid and comprehensive changes across all facets of society to mitigate the effects of global warming and limit it to 1.5 °C by the end of this century. This underscores the need to address environmental challenges and the broader spectrum of social and economic issues ( Allen *et al.*, 2018).

### 1.1 Education for sustainable development

Nearly 200 nations expanded the scope from the Millennium Development Goals (MDGs) to the Sustainable Development Goals (SDGs) at the 2015 United Nations Summit ( WHO, 2015). To effectively implement these goals globally, most of the world's population must grasp the holistic concept of SDGs, recognize their accountability for their actions, and actively contribute to the vision of a sustainable future ( Glavič, 2020). Therefore, enhancing existing knowledge, values, and attitudes regarding sustainability among individuals is paramount, enabling them to make behavioral changes to support a sustainable future. The younger generation, in particular, should possess sufficient sustainability literacy to assume responsibility for their actions and actively participate in shaping a sustainable future. Education plays a pivotal role in realizing these sustainability objectives. Instilling a foundational understanding of sustainable development among youth is crucial for monitoring their evolving perceptions and behaviors related to consumption and sustainability literacy ( Chen *et al.*, 2022).

Education for Sustainable Development (ESD) represents a holistic and transformative educational approach, addressing content, learning outcomes, pedagogy, and the learning environment. This underscores the importance of equipping individuals with the knowledge, skills, values, and attitudes that empower them to actively engage in collaborative efforts for sustainable development initiatives ( Lozano *et al.*, 2017). Therefore, Education for Sustainable Development (ESD) is a lifelong learning process recognized as a crucial catalyst for achieving all 17 Sustainable Development Goals, notably contributing to Goal 4 on Education ( Smaniotto *et al.*, 2020). Individual sustainability literacy can be defined as the mindset and capabilities required to maintain sustainable actions and contribute to establishing a sustainable future ( Diamond & Irwin, 2013). Raising public awareness of sustainability and fostering the necessary skills for effective change is crucial in shaping consumers' perceptions of their choices and enhancing their active engagement in research and development endeavors for scientific progress ( Katsaliaki & Mustafee, 2014).

Furthermore, ESD is important in three key SDGs: Goal 4 (Quality Education), Goal 12 (Responsible Consumption and Production), and Goal 13 (Climate Action). Aligned with Target 4.7: the objective for 2030 is to ensure that all learners acquire the knowledge and skills necessary to promote sustainable development. This includes fostering education for sustainable development and encouraging the adoption of sustainable lifestyles. Specifically, Target 12.8 highlights the importance of ensuring that individuals worldwide have access to relevant information and awareness about sustainable development, enabling them to make informed choices that align with environmental sustainability. Moreover, Target 13.3 underscores the need to enhance education, understanding, and institutional capacity to address climate change. This includes efforts in mitigation, adaptation, impact reduction, and early warning systems, reinforcing the role of education in building climate resilience. ( Diamond & Irwin, 2013; Katsaliaki & Mustafee, 2014; Lozano *et al.*, 2017; Smaniotto *et al.*, 2020).

### 1.2 Digital science communication

Information and Communication Technology (ICT) has effectively transformed our world into a global village driven by globalization. Within this evolving landscape, education, ICT, and innovation in science and technology have emerged as the three foundational pillars of the knowledge society ( Malik, 2018). The human brain benefits greatly from visual data representations to comprehend complex scenarios. Visual materials such as diagrams, schematics, drawings, and, more recently, photographs, films, and satellite images have been instrumental in conveying scientific discoveries since the early days of modern science ( Franconeri *et al.*, 2021). The well-known saying, "A picture is worth a thousand words," underscores the comprehensive understanding that audiences can gain from visual materials ( Bucchi & Saracino, 2016; Siricharoen, 2013). In digital communication, knowledge generation and the dissemination of scientific information are becoming increasingly inclusive and socially oriented. To effectively engage with the non-scientific community, science communicators and educators employ the concept of "edutainment," especially with the availability of new technologies in modern higher education institutions ( Anikina & Yakimenko, 2015).

In theory, outreach science communication encompasses knowledge sharing, raising awareness, and educating non-scientists, as seen in science journalism and citizen science initiatives ( Bonney *et al.*, 2016; Dickel & Franzen, 2016; Illingworth & Allen, 2020). While simplifying intricate scientific information to make it accessible to a lay audience is commendable, it is essential to strike a balance, as excessive simplification can lead to misconceptions about complex concepts and study limitations ( Kloepper, 2017). According to Fontaine *et al.* (2019), 70% of the public seeks scientific information on the Internet, and digitalization empowers scientists and individuals to communicate transparently about their research. A cross-media approach employing digital multimedia tools offers a powerful means of presenting the dynamic nature of scientific topics in engaging and understandable ways ( Rigutto, 2017). Multiple case studies in domains including healthy weight management, tobacco control, and vaccination uptake demonstrated that Digital Media for behavior change encompasses three primary methods: digital media interventions, formative research employing digital media, and digital media for conducting evaluations. Hence, digital (online visual) science communication involves transferring scientific knowledge through informative graphical content and animated videos delivered via educational multimedia platforms such as websites, mobile applications, edutainment games, broadcasting channels, and video communications, reaching a diverse audience ( Evans *et al.*, 2022).

### 1.3 Integration of information and communication technology in science education

For many years, educational courses were structured around textbooks, with teaching primarily delivered through lectures and presentations, complemented by tutorials and learning activities designed to reinforce and practice the subject matter ( Yusuf *et al.*, 2013). Traditional education paradigms have relinquished their exclusive control over the primary content of student learning. This content has transcended geographical boundaries and is now disseminated within the boundless realm of the internet ( Fauville *et al.*, 2013). Therefore, conventional educational institutions no longer hold exclusive control over knowledge, as we now exist and acquire knowledge within the virtual knowledge ecosystem. With this virtual knowledge ecosystem, information undergoes continuous amalgamation, enrichment, and evolution ( Breck, 2006). Traditional teaching underscored content delivery, while present-day educational contexts increasingly prioritize curricula fostering competence and enhanced performance. Present-day curricula strongly emphasize capabilities and the practical utilization of information and communication technology over mere theoretical knowledge of such technology ( Breck, 2006; Fauville *et al.*, 2013; Yusuf *et al.*, 2013).

 Information and communication technology (ICT) is an integral component of today's world, necessitating adjustments in culture and society to address the demands of the knowledge age. The adoption of ICT has brought about fundamental shifts in the practices and procedures across a broad spectrum of fields, encompassing economics, bureaucracy, social communication, and civil administration, including science education ( Nadda, 2022). Educational research in Japan, Canada, and Nigeria has revealed that heightened exposure to educational ICT integration within the curriculum positively affects student academic performance, notably in science-based subjects ( Laronde *et al.*, 2017). Hence, ICT assumes the role of a transformative agent within higher education. On the other hand, growing evidence of climate change, global warming, and financial and socio-cultural concerns are elevating the importance of sustainability within higher education curricula ( Leal Filho, 2000). The theme of 'Digital Science Communication, ICT, and Education for Sustainable Development (ESD) constitutes a three-fold strategy, encompassing the instruction of sustainable development, the utilization of digital technologies, including ICT, and the behavior change through innovative and interactive pedagogical methods ( Ricard *et al.*, 2020).

### 1.4 Pandemic impact on sustainable development

The COVID-19 pandemic is one of this century's deadliest biological threats to humanity, rivaling the 1918 Flu pandemic ( Khan *et al.*, 2020). Its impacts on human well-being and pursuing Sustainable Development Goals are profoundly distinct. Additionally, the pandemic introduces interference with the attainment of Sustainable Development Goals, which form the vital foundation for addressing global challenges such as hunger, inequality, natural resource conservation, and bolstering climate change adaptation for the long-term protection of our planet for future generations ( Hörisch, 2021). In response to COVID-19, the World Health Organization (WHO) and nearly all national governments implemented preventive measures like social distancing, lockdowns, and stay-at-home directives. These measures substantially impacted individuals' daily routines and behaviors ( Marroquín *et al.*, 2020).

Consequently, numerous researchers explored the multifaceted impacts of COVID-19 on various aspects of life. These impacts on sustainability were immense and posed significant challenges to the global pursuit of Sustainable Development Goals. They encompassed the closure of schools, effects on mental well-being, travel restrictions, alterations in work patterns, labor shortages, home isolation, and many more impacts ( Azevedo *et al.*, 2021). According to the United Nations, school closures affected 91% of students worldwide, and long-term home isolation heightened the risk of mental health issues among students ( Lee, 2020).

Paradoxically, the pandemic also yielded unexpected positive outcomes concerning Sustainable Development Goals. For instance, technology played a pivotal role in bridging gaps in the education system in Ireland, enabling continuous education for students through digital communication channels, thereby contributing to the realization of quality education as per SDG 4 ( Mohan *et al.*, 2020). The usefulness of digital tools is becoming widespread, affecting daily areas of life, including social, economic, and political aspects. The COVID-19 pandemic has made having a digital presence even more critical ( Davis *et al.*, 2014; Sá *et al.*, 2022). Behavioral change can be defined as the sustained alteration of individual habits and lifestyles ( Alturki & Aldraiweesh, 2021). WHO and various national governments engaged in health education and promoted behavioral adaptations to the pandemic through digital science communication using multimedia.

This approach also contributed to achieving quality healthcare in alignment with SDG 3. These initiatives demonstrated that distance learning through digital communication channels could effectively surmount the physical absence of teachers or instructors, successfully fostering behavioral change among recipients ( Pedersen *et al.*, 2021; Wang & Huang, 2021).

### 1.5. Purpose of the study

This scoping review examines how digital science communication enhances learners' sustainability literacy within the broader Education for Sustainable Development (ESD) framework and the 2030 Agenda. The review explores the impacts and obstacles associated with using digital tools to foster sustainability-related knowledge, values, and behaviors among learners across diverse educational and geographical settings. Sustainability literacy refers to the knowledge, skills, and values that empower individuals to make informed decisions and take responsible actions that advance environmental protection, economic viability, and social equity. These competencies are considered critical for achieving the Sustainable Development Goals (SDGs), particularly targets 4.7, 12.8, and 13.3 ( UNESCO, 2020). The SDGs are recognized as a universal framework for promoting peace and prosperity for humanity and the planet, both now and in the future ( Agbedahin, 2019; De Jong & Vijge, 2021; Griffiths, 2020).

This study adopts a scoping review approach to systematically map existing research across different levels of education (Population), focusing on the role of digital science communication tools and practices (Concept), within the context of Education for Sustainable Development after the adoption of the MDGs in 2000 (Context). The primary objectives of this review are:

▪ To identify which digital tools have been applied to promote sustainability literacy and how they are being used;
▪ To assess the educational levels, fields, and locations in which these approaches have been implemented;
▪ To explore the impacts and obstacles of using digital science communication in sustainability education.
▪ To highlight knowledge gaps and inform future empirical studies and policy interventions.

## 2. Materials and methods

Given the exploratory nature of the topic, the diversity of digital communication tools, and the wide range of educational contexts in which they are applied, a scoping review approach was deemed appropriate to systematically map the extent, range, and characteristics of existing research. This methodology is well-suited for identifying knowledge gaps, clarifying conceptual boundaries, and informing future empirical studies in an emerging and fragmented field lacking prior comprehensive synthesis.

Accordingly, this review focuses on peer-reviewed scientific articles published between 2000 and 2023 that examine digital science communication or information and communication technologies (ICT) in Education for Sustainable Development (ESD), particularly on sustainability literacy. The post-2000 period was chosen to align with adopting the Millennium Development Goals (MDGs), which marked a global shift toward integrated development frameworks.

The research team discussed and drafted the study protocol by PRISMA-ScR guidelines; however, a formal review protocol was not registered or published beforehand. Despite this, the scoping review was conducted with strict adherence to the PRISMA Extension for Scoping Reviews (PRISMA-ScR) guidelines, thereby ensuring transparency, methodological rigor, and reproducibility at all stages of the review process ( Tricco *et al.*, 2018; Zimmerman *et al.*, 2023).

The study applied principles of qualitative data analysis and synthesis to achieve its aims, drawing on established techniques for qualitative evidence synthesis to identify and characterize the most relevant and representative studies in the field ( Sandelowski & Barroso, 2007).

### 2.1 Formulate *Guiding Research Q*uestion

This scoping review addresses the following guiding research questions. What are the impacts and obstacles associated with employing digital science communication to enhance learners' sustainability literacy and promote the adoption of more sustainable behaviors and practices?

 Aspects related to this research question include: In which research environments, including the academic level and fields of study, were the studies conducted, and which countries served as the primary locations for this research? In what ways can the approach of digital science communication or ICT tools potentially shape learners' outlooks, practices, and behaviors toward greater sustainability? How does the utilization of digital science communication impact learners' sustainability literacy? What obstacles and variables are linked to applying these methods to enhance learners’ sustainability literacy? What evidence exists regarding the contribution of Digital Science Communication to achieving SDG targets 4.7, 12.8, and 13.3 in the context of sustainability literacy? 

### 2.2 Comprehensive initial search

**Figure 1.**
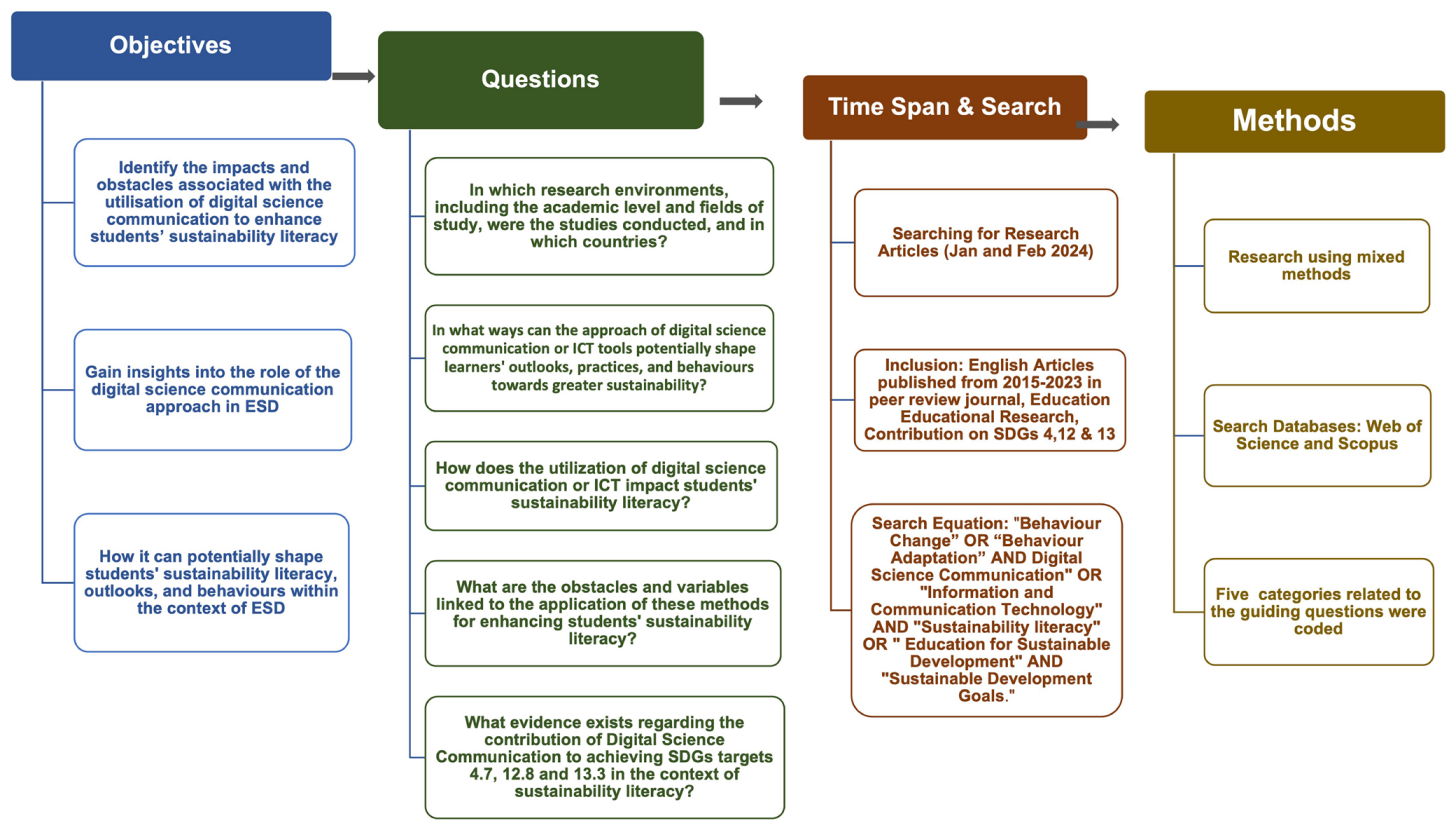
Parameters defined in the study.

A comprehensive initial search was conducted systematically and sequentially, emphasizing the broad retrieval of relevant articles to establish parameters related to topics, populations, timelines, and research methods ( Figure 1). This approach aligns with the principles for robust evidence synthesis as guided by the PRISMA Extension for Scoping Reviews (PRISMA-ScR), ensuring methodological rigor and transparency ( Tricco *et al.*, 2018).

**Figure 2.**
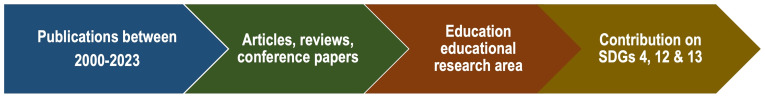
Inclusion criteria.

The information-gathering process focused on selected databases, namely the Web of Science (WOS) and Scopus. The article search and selection period spanned from January to February 2024 and was conducted using the Virtual Private Network (VPN) of the University of Lisbon. Subsequently, inclusion criteria were defined, as described in Figure 2.

A search strategy employing a combination of specific keywords and Boolean operators (e.g., AND, OR), customized to the databases' requirements. The complete and reproducible search strings used for each database are provided in the extended data. The search query included terms such as “Behaviour Change” OR “Behaviour Adaptation,” AND “Digital Science Communication,” OR “Information and Communication Technology”, AND “Sustainability literacy,” OR “Education for Sustainable Development”, AND “Sustainable Development Goals” in either the title or in the keywords. The time frame of 2000 to 2023 was selected to align with the launch of the Sustainable Development Goals (SDGs), which succeeded the Millennium Development Goals (MDGs), and to correspond with the global push for Education for Sustainable Development since then.

### 2.3. Initial study selection

**Figure 3.**
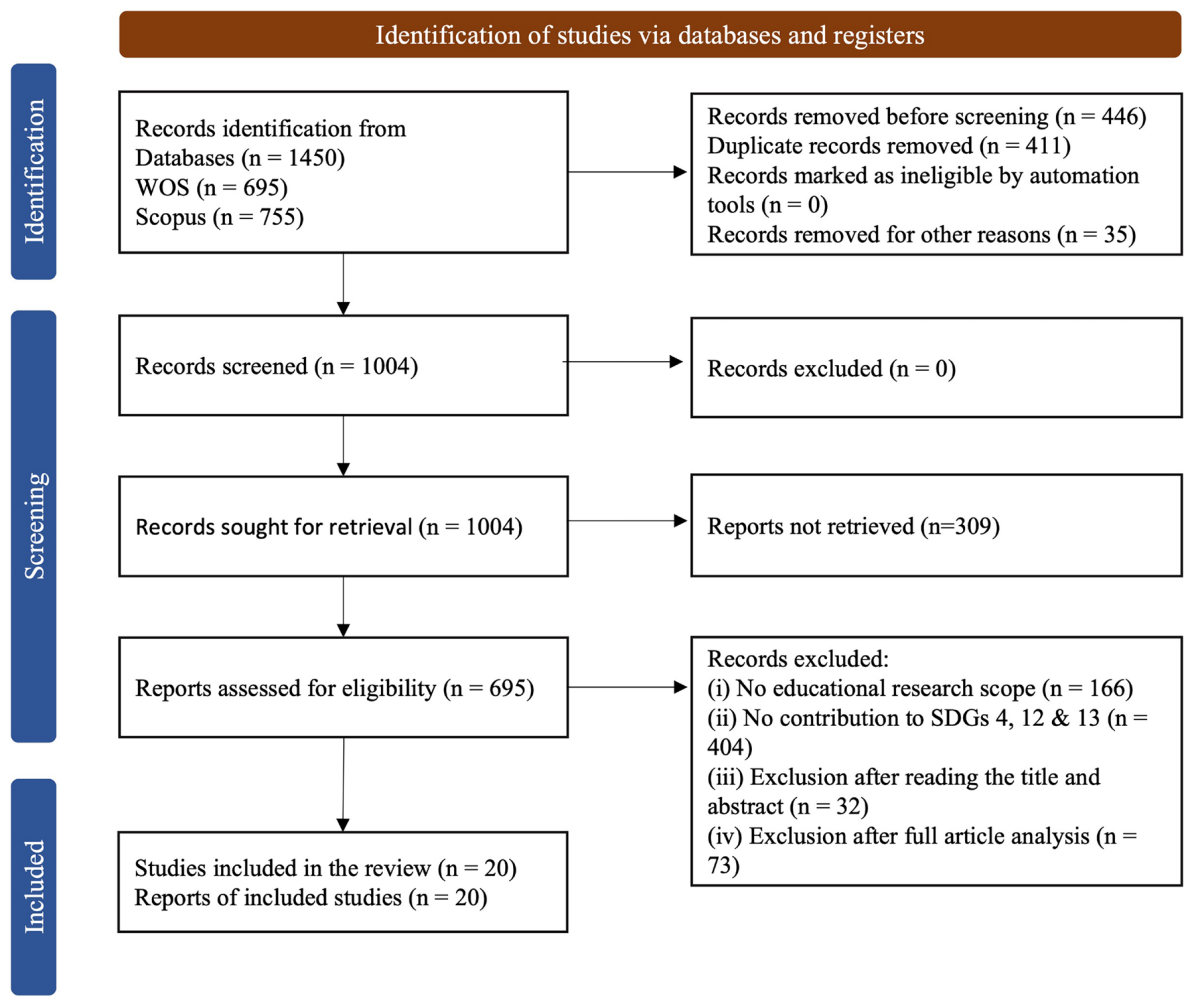
Schematic representation of the review process (Adhering to the PRISMA Protocol PRISMA-ScR ( Tricco *et al.*, 2018).

This scoping review was conducted by the PRISMA Extension for Scoping Reviews (PRISMA-ScR) guidelines ( Tricco *et al.*, 2018) to ensure methodological rigor, transparency, and reproducibility. A visual summary of the search and selection process is provided in Figure 3. Initial comprehensive searches were carried out using two major bibliographic databases: Web of Science (WoS) and Scopus, with the final search completed in February 2024. The full search strategies, including Boolean logic and keyword strings, are detailed in Section 2.2. The search yielded a total of 1,450 records, comprising 695 from WoS and 755 from Scopus. After exporting all records, a preliminary pre-screening process excluded 446 entries. This included 411 duplicates, identified and removed using EndNote, and 35 non-eligible records, such as conference abstracts that did not meet the inclusion criteria for full publication. This initial exclusion process primarily affected records from Scopus or duplicate entries where the Scopus version was discarded, ensuring the full 695 WoS records remained in the deduplicated dataset.

This resulted in 1,004 unique records entering the formal screening phase. Of these 1,004 unique records, the entire set of 695 records identified initially from Web of Science remained intact after the preliminary pre-screening. Upon further comparison, it became evident that the Web of Science (WoS) database offered two key methodological advantages that aligned closely with the aims and thematic focus of this review: (1) WoS provides a more granular subject classification, including a distinct disciplinary category for ‘Education Educational Research,’ which lacks a direct equivalent in Scopus; and (2) WoS supports direct filtering by specific Sustainable Development Goals (SDGs), namely, SDG 4 (Quality Education), SDG 12 (Responsible Consumption and Production), and SDG 13 (Climate Action), a functionality not currently available in Scopus. These features enabled a more precise and consistent application of the eligibility criteria. Accordingly, while both databases contributed to the initial mapping of the literature landscape, the primary screening and selection process was conducted exclusively on the WoS records.

The 695 unique WoS records (a subset of the 1,004 total unique records) were further refined using the platform’s automated filtering tools. Applying the disciplinary filter for ‘Education Educational Research’ reduced this dataset to 529 records. These were then further filtered to retain only those indexed under SDGs 4, 12, or 13, resulting in 125 records eligible for subsequent manual screening of abstracts, and eventual full-text review and thematic synthesis. The filtering process was conducted by reviewer Aung, T. S., following predefined inclusion criteria aligned with the conceptual focus on sustainability competence, with particular emphasis on sustainability literacy and adopting sustainable lifestyles. 

### 2.4. Final selection and data charting

Titles and abstracts of the 125 records were initially screened, resulting in 93 articles retained for full-text review. A subsequent full-text assessment led to the exclusion of 73 articles that did not sufficiently align with the research questions or lacked clear contributions to the dimensions of sustainability competence. This process yielded a final sample of 20 articles selected for in-depth analysis. 

The detailed review involved systematically extracting evidence relevant to the guiding research questions. To support this, an analysis matrix was developed encompassing the following elements:

1. Publication year
2. Bibliographic details
3. Academic settings, research fields, and geographical locations
4. Main themes and ideas addressed
5. Connections between the main themes and research questions
6. Textual segments containing relevant evidence
7. Indications of contributions to SDG targets 4.7, 12.8, and 13.3 relating to sustainability literacy

The complete analysis matrix was deposited in the Zenodo platform. (See extended data). The data from the 20 included studies were charted using a standardized extraction matrix developed in Microsoft Excel, which was pilot-tested on a sample of three articles to ensure clarity and consistency of categories. The first reviewer conducted Data charting independently (Aung, T. S.), and the extracted entries were cross-checked for consistency across the defined categories. NVIVO 12 organized and coded qualitative data using five thematic categories aligned with the research questions. No authors of primary studies were contacted for additional data, as the full texts provided sufficient detail for extraction and synthesis. 

### 2.5. Summary of findings from selected studies using qualitative techniques

A qualitative analysis was performed based on the information in the analysis matrix. Five categories related to the guide questions were coded using NVIVO 12 software. One category was associated with each question.

- Academic settings, research fields, and locations: Gather information regarding the educational level, field of study, and geographic locations where the studies were conducted.
- Digital science communication tools and approaches: Identify the various tools utilized to enhance learners' sustainability literacy and how these tools influence changes in learners' behaviors to promote greater sustainability.
- Impacts of digital science communication: Compiles the observed impacts resulting from the application of digital science communication in improving learners' sustainability literacy.
- Obstacles and variables associated with the application: Recognises and examines the challenges and variables associated with implementing digital science communication approaches to enhance sustainability literacy among learners.
- Evidence on achieving designated SDG targets: Collects the available evidence demonstrating the contribution of digital science communication to attaining SDG targets 4.7, 12.8, and 13.3.

These classifications were employed during the analysis and interpretation phase, resulting in a distribution summarised in Table 1.

**Table 1.** Findings from selected studies, categories, and references.

Categories	Numbers	References
Academic Settings, Research Fields, and Locations	20	Aguayo & Eames; 2017; Aguayo & Eames, 2023; Altomonte *et al.*, 2016; Andriopoulou *et al.*, 2022; Colás-Bravo & Quintero-Rodríguez, 2023; Delgado-Plaza *et al.*, 2021; Fjællingsdal & Klöckner, 2019; Gatti *et al.*, 2019; Hilty & Huber, 2018; Horng *et al.*, 2022; Istenic Starcic *et al.*, 2018; Kabadayi *et al.*, 2022; Liu *et al.*, 2019; Martínez-Borreguero *et al.*, 2020; Mercer *et al.*, 2017; Robin *et al*., 2017; Rodríguez-Loinaz *et al.*, 2022; Shelton *et al.*, 2016; Tsai, 2018; Veronica & Calvano, 2020
Digital Science Communication Tools and Approaches	20	Aguayo & Eames; 2017; Aguayo & Eames, 2023; Altomonte *et al.*, 2016; Andriopoulou *et al.*, 2022; Colás-Bravo & Quintero-Rodríguez, 2023; Delgado-Plaza *et al.*, 2021; Fjællingsdal & Klöckner, 2019; Gatti *et al.*, 2019; Hilty & Huber, 2018; Horng *et al.*, 2022; Istenic Starcic *et al.*, 2018; Kabadayi *et al.*, 2022; Liu *et al.*, 2019; Martínez-Borreguero *et al.*, 2020; Mercer *et al.*, 2017; Robin *et al*., 2017; Rodríguez-Loinaz *et al.*, 2022; Shelton *et al.*, 2016; Tsai, 2018; Veronica & Calvano, 2020
Impacts of Digital Science Communication	20	Aguayo & Eames; 2017; Aguayo & Eames, 2023; Altomonte *et al.*, 2016; Andriopoulou *et al.*, 2022; Colás-Bravo & Quintero-Rodríguez, 2023; Delgado-Plaza *et al.*, 2021; Fjællingsdal & Klöckner, 2019; Gatti *et al.*, 2019; Hilty & Huber, 2018; Horng *et al.*, 2022; Istenic Starcic *et al.*, 2018; Kabadayi *et al.*, 2022; Liu *et al.*, 2019; Martínez-Borreguero *et al.*, 2020; Mercer *et al.*, 2017; Robin *et al*., 2017; Rodríguez-Loinaz *et al.*, 2022; Shelton *et al.*, 2016; Tsai, 2018; Veronica & Calvano, 2020
Obstacles and Variables Associated with the Application	9	Aguayo & Eames; 2017; Aguayo & Eames, 2023; Altomonte *et al.*, 2016; Colás-Bravo & Quintero-Rodríguez, 2023; Delgado-Plaza *et al.*, 2021; Fjællingsdal & Klöckner, 2019; Hilty & Huber, 2018; Martínez-Borreguero *et al.*, 2020; Shelton *et al.*, 2016
Evidence on Achieving Designated SDGs Targets for ESD	20	Aguayo & Eames; 2017; Aguayo & Eames, 2023; Altomonte *et al.*, 2016; Andriopoulou *et al.*, 2022; Colás-Bravo & Quintero-Rodríguez, 2023; Fauville *et al.*, 2013; Franconeri *et al.*, 2021; Griffiths, 2020; Hilty & Huber, 2018; Illingworth & Allen, 2020; Istenic Starcic *et al.*, 2018; Leal Filho, 2000; Marroquín *et al.*, 2020; Martínez-Borreguero *et al.*, 2020; Rigutto, 2017; Robin *et al.*, 2017; Rodríguez-Loinaz *et al.*, 2022; Shelton *et al.*, 2016; Tsai, 2018; Veronica & Calvano, 2020

## 3. Results

The outcomes of this investigation are situated within the context of the five categories presented as follows ( 3.1 to 3.5).

### 3.1 Academic settings, research fields, and locations

In detail, four studies were conducted at the primary level, encompassing 30 participants in New Zealand, 50 pupils in Italy, 60 in the UK, and 6 participants in France ( Aguayo & Eames, 2023; Mercer *et al.*, 2017; Robin *et al.*, 2017; Veronica & Calvano, 2020). 

Additionally, three studies were carried out at the secondary level, involving 81 participants in Spain, 153 participants in Greece, and 68 participants in Taiwan ( Andriopoulou *et al.*, 2022; Martínez-Borreguero *et al.*, 2020; Tsai, 2018). 

Furthermore, eight studies were conducted at the tertiary education level, with 360 participants in China, 847 participants in Slovenia, 32 and 405 participants in the UK, 127 and 567 participants in Taiwan, and 80 and 54 participants in business schools of the university in Switzerland, respectively ( Altomonte *et al.*, 2016; Gatti *et al.*, 2019; Hilty & Huber, 2018; Horng *et al.*, 2022; Istenic Starcic *et al.*, 2018; Liu *et al.*, 2019; Mercer *et al.*, 2017; Tsai, 2018).

Three studies focused on the professional training domain, with 143 participants in the Czech Republic, 100 in Spain, and 223 in the US ( Delgado-Plaza *et al.*, 2021; Kabadayi *et al.*, 2022; Shelton *et al.*, 2016).

Moreover, one study was conducted at the community level, involving 24 local community members from the target community in Chile ( Aguayo & Eames, 2017). 

Lastly, three studies involved mixed levels of education, comprising 504 participants in Spain, 56 in Norway, and 221 in Greece, the UK, and Spain combined ( Colás-Bravo & Quintero-Rodríguez, 2023; Fjællingsdal & Klöckner, 2019; Rodríguez-Loinaz *et al.*, 2022). 

**Figure 4.**
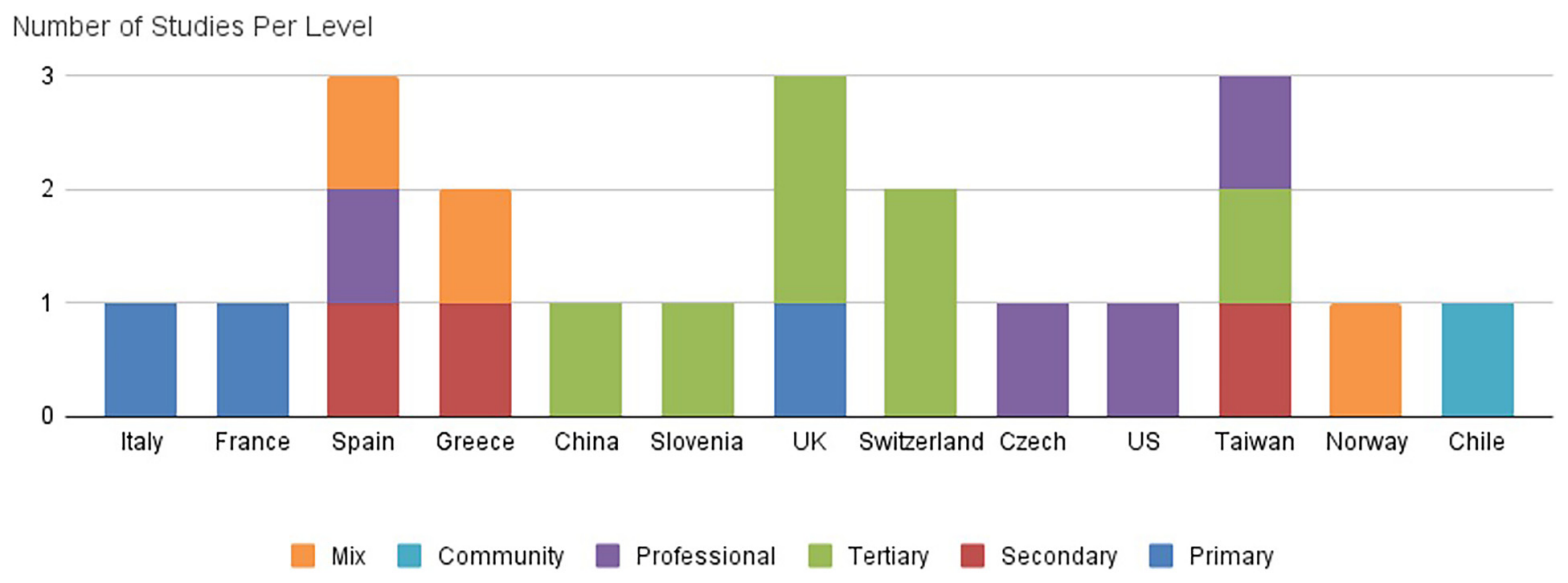
Selected Digital Science Communications Studies Conducted in Different Countries and Corresponding Levels.

Notably, all the detailed findings of the digital science communication studies carried out after adopting the SDGs in 2015, even though we included the review starting from 2000 ( Figure 4). 

Based on the data analysis, numerous studies have concentrated on utilizing digital science communication to improve sustainability literacy, with a notable concentration in European countries. A smaller number of studies were conducted in Australia, Asia, South America, and North America, particularly since the adoption of the UN SDGs in 2015. Moreover, there were significant variations in sample sizes among the studies. Most study samples were from education, although a few studies involved tourism, business, and development representatives.

### 3.2 Digital science communication tools and approaches

In the primary education domain, these studies explored students' experiences transitioning from virtual reality (VR) to natural marine environments, focusing on the educational impacts of immersive technologies like VR, augmented reality (AR), extended reality (XR), and 3D visualization ( Aguayo & Eames, 2023). Another study introduced educational interventions, including a serious game and an explanatory video, aiming to address the critical issue of marine waste and its impact on marine species. These initiatives aimed to improve students' understanding of marine ecosystems, raise awareness about the environmental consequences of marine pollution, and promote the use of a specialised website for community-driven Education for Sustainable Development (ESD) projects ( Mercer *et al.*, 2017; Veronica & Calvano, 2020). This educational approach involves ongoing learning and problem-solving ( Robin *et al.*, 2017).

Three distinct digital science communication approaches were employed in the secondary education domain. The first study utilized resources like WebQuests and video games within a discovery learning model to investigate how these ICT-based interventions impact secondary school students' sustainability content, encompassing cognitive and affective aspects ( Martínez-Borreguero *et al.*, 2020). The second study focused on Digital Storytelling (DST) as an instructional approach in a non-formal learning setting, specifically at the Hellenic Centre for Marine Research. It aimed to explore the effectiveness of DST in shaping positive attitudes among high school students toward topics related to plastic pollution and marine trash in the Mediterranean ( Andriopoulou *et al.*, 2022). Lastly, another study introduced the Socio-Scientific Issues of Online Argumentation Pattern (SOAP) as a pedagogical approach, empowering students to engage effectively in online arguments about Socio-Scientific Issues (SSI) ( Tsai, 2018).

In the scope of tertiary education, several studies tested different aspects of digital science communication tools for sustainability literacy. One study aimed to enhance environmental ethics education using virtual reality techniques. Another focused on students' perceptions through their social impact on sustainable development awareness and behaviors. Additionally, a study explored the potential of interactive e-learning and m-learning courses in environmental disciplines to promote sustainable development education. Another study identified specific course content that engages students in sustainable development, particularly those in ICT-related programs. Lastly, a Swiss business school study investigated experiential learning through simulation games to teach sustainability concepts to business-specialized students ( Altomonte *et al.*, 2016; Gatti *et al.*, 2019; Hilty & Huber, 2018; Istenic Starcic *et al.*, 2018; Liu *et al.*, 2019). Additionally, a study with university students in tourism examined how using sharing economy platforms affected their learning and attitudes toward sustainability ( Horng *et al.*, 2022). 

In the professional training domain, various studies focused on professionals like kindergarten principals and pre-service teachers. For instance, one study examined how kindergarten principals use technology, specifically digital science communication, to support sustainability-focused learning ( Kabadayi *et al.*, 2022). Another study explored online platforms and digital tools for long-term sustainable development after the COVID-19 pandemic ( Delgado-Plaza *et al.*, 2021). Lastly, a study with pre-service teachers used digital storytelling to enhance their understanding of sustainability science in online courses ( Shelton *et al.*, 2016).

Another case study explores how digital science communication is used for community-based learning about sustainable development in southern Chile. It investigates the environmental problems related to the degradation of a lake and the broader socio-ecological impacts caused by this issue ( Aguayo & Eames, 2017).

Some studies adopted a mixed model of sample groups. One study used quantitative methods to explore informal learning on YouTube for sustainable development programs, looking at age, gender, and education level as factors. They used statistical techniques to analyze the differences in informal learning ( Colás-Bravo & Quintero-Rodríguez, 2023). Another study aimed to assess the effectiveness of the online Eco platform by the US Department of Education in raising awareness about ecosystems ( Fjællingsdal & Klöckner, 2019). The last study, which also used a mixed sample of groups, explored how ICT-supported citizen science can enhance transformative teaching in education for sustainable development ( Fletcher, 2017).

### 3.3 Impacts of digital science communication

In the primary education scope, the first study discovered that most students enjoyed learning through virtual reality (VR) and recognized its potential for innovative education, especially when it included engaging elements like missions. It also showed that extended reality (XR) technologies could significantly improve students' understanding of marine ecology, as evidenced by better post-test results. Furthermore, the research highlighted how augmented reality (AR) could connect students' learning experiences across different contexts, emphasizing the role of digital science communication in enhancing sustainability literacy ( Aguayo & Eames, 2023). In the second study, a serious game aimed to improve ocean literacy, focusing on the biodiversity of the Apulian and Mediterranean seas to raise children's awareness of marine life preservation. The study confirmed that children could acquire knowledge effectively through new technologies and serious games ( Veronica & Calvano, 2020). The third study enhanced students' understanding of sustainability. It equipped them with problem-solving and design-thinking skills, fostering social responsibility and sustainable attitudes that could address real-life issues beyond the classroom. Understanding the unique characteristics and needs of the local community was crucial for providing impactful and culturally relevant learning experiences ( Mercer *et al.*, 2017).

In secondary education, the first study evaluated the impact of video games and WebQuests on students, focusing on their knowledge and attitudes toward sustainability. The results showed that these ICT educational activities effectively promoted learning, particularly among students who engaged more with video games ( Martínez-Borreguero *et al.*, 2020). The second study, a pioneering case, used digital storytelling to address plastic marine pollution, significantly improving students' performance. This approach raised awareness and transformed attitudes towards marine litter issues ( Andriopoulou *et al.*, 2022). In the third study, the Socio-Scientific Issues of Online Argumentation Pattern (SOAP) facilitated interaction among educators and students, enhancing student engagement and participation ( Tsai, 2018).

Research in the tertiary education domain indicates that virtual reality experiences and practical courses can generate interest, improve the learning experience, and promote environmental ethics and practical skills. These experiences enhance cognitive learning and cultivate a strong sense of ecological responsibility, allowing participants to apply their knowledge and skills to real-world environmental challenges ( Liu *et al.*, 2019). Additionally, online social media plays a significant role in higher education for sustainable development, fostering connections and resources that support sustainability efforts. Integrating educational technology into higher education is vital, especially when teaching systems thinking in merging ICT and sustainable development disciplines ( Altomonte *et al.*, 2016; Hilty & Huber, 2018; Istenic Starcic *et al.*, 2018). A study by a university business school shows that students understand the importance of sustainability across an enterprise, making business simulations a valuable tool for sustainability literacy ( Gatti *et al.*, 2019). In the tourism sector, university students showed increased awareness of sustainability-related topics like environmental concerns and energy efficiency when using online platforms ( Horng *et al.*, 2022). 

The professional training domain involving kindergarten principals and educators highlights the importance of sustainable learning practices for children, emphasizing responsible resource use in areas like materials, food, energy, and water. These studies indicate that ICT has substantial potential to enhance the learning process in sustainable education ( Delgado-Plaza *et al.*, 2021; Kabadayi *et al.*, 2022). Another study with pre-service teachers also suggested the incorporation of digital storytelling, focusing on online sustainability science courses; digital storytelling introduces the intricate concepts of sustainability science to individuals without a specialized background in the field ( Shelton *et al.*, 2016).

Another case study in Chile offers real-world proof of how ICT can improve community-based Education for Sustainable Development (ESD). It involved creating a theoretical framework that combined learning theories from ICT and ESD, and findings revealed that digital science communication effectively fostered deep and transformative learning, locally rooted ecological literacy, active engagement, and the adoption of sustainable living principles and practices within certain community members ( Aguayo & Eames, 2017).

In the mixed-level education study domain, it was found that digital science communication impacted sustainability literacy. The study using the Eco online platform showed positive results, indicating that the Eco platform effectively enhanced participants' environmental literacy and systems thinking. Eco was found to strengthen awareness of the ecological impact of human actions on ecosystems. This suggests that Eco could be a valuable tool for improving environmental consciousness related to ecosystems, with recommendations for future use ( Fjællingsdal & Klöckner, 2019). Another study by Rodríguez-Loinaz *et al*. in 2022 demonstrated that innovative ICT tools empowered teachers to engage students in citizen science activities tailored to their local environment.

### 3.4 Obstacles and variables associated with the application

In the primary education domain, the author of the first study recommends that virtual reality (VR) should enhance traditional primary education study methods rather than replace them entirely. This approach helps children develop a systemic worldview, expanding their horizons, even at a young age. However, it has been noted that children may initially focus on using the VR game rather than fully grasping sustainable development concepts. In addition, the price of those VR headsets and WIFI access can also be an obstacle to using them more widely ( Aguayo & Eames, 2023). 

In secondary education, it is acknowledged that changing environmental attitudes and behaviors in a single classroom session can be challenging, suggesting extended interventions with active methodologies to foster positive emotions and beliefs. Another study indicates that ICT positively impacts academic progress, with video games more appealing to boys than girls (gender bias). In addition, video games covering some sustainability curricula are lacking, thus often requiring the adaptation of existing video game content to meet specific educational needs ( Martínez-Borreguero *et al.*, 2020).

At the tertiary education level, digital science communication tools like e-learning courses can enhance student-centered, in-depth learning, especially in sustainable design, when combined with hands-on experiences and diverse teaching methods. However, it is essential to note that online tools cannot fully replace in-person instruction. Integrating systems thinking, crucial for teaching sustainability, presents a pedagogical challenge when merging ICT and sustainable development ( Altomonte *et al.*, 2016; Hilty & Huber, 2018).

In the professional training domain, age does not significantly influence technology adoption for virtual teaching, and ESD directly contributes to achieving SDGs ( Delgado-Plaza *et al.*, 2021). Another Study with pre-service teachers found a fascinating point regarding the design of the online course. Participants who raised concerns about accountability noted that they could achieve favorable results in the interactive quizzes even without comprehensively viewing all the assigned content. In addition, students mentioned difficulties such as the inability to rewind or fast-forward within the videos ( Shelton *et al.*, 2016).

Today, we are using more technology but also growing distant from our local natural places, which are crucial for our environment. To address this, the study at the community level aimed to use technology to reconnect people with nature and each other in culturally meaningful ways ( Aguayo & Eames, 2017).

The studies with different academic levels revealed exciting findings. Gender did not show significant differences, but age did. People between 50 and 60 responded differently from those below 30, possibly due to younger individuals being more adapted to technology. This highlights the need for further research to explore the technology gap between age groups and consider factors like economic status, cultural background, or geographic region ( Colás-Bravo & Quintero-Rodríguez, 2023). In the study with the Eco platform, all participants were male despite varying education levels. Future research should aim for greater gender diversity to avoid potential bias in research outcomes ( Fjællingsdal & Klöckner, 2019). 

### 3.5 Evidence on achieving designated sdgs targets 4.7, 12.8 & 13.3

Mixed reality (MR) immersive learning positively changes children's behavior in primary education, with increased environmental awareness and eco-friendly practices. Serious game interventions at this level also sparked fresh insights and improved student habits, as noted by both students and teachers. Additionally, serious games help children understand prospective thinking ( Aguayo & Eames, 2023; Veronica & Calvano, 2020).

In secondary education, Information and Communication Technology activities enhanced sustainability literacy. Digital storytelling engaged students and advanced scientific education and environmental literacy in a marine pollution context. Similarly, socio-scientific online argumentation patterns fostered social responsibility and sustainability attitudes while connecting classroom knowledge to real-life situations ( Andriopoulou *et al.*, 2022; Martínez-Borreguero *et al.*, 2020; Tsai, 2018).

At the tertiary level, virtual reality and hands-on courses effectively interested and engaged learners in environmental action. Social media played a pivotal role in supporting sustainability discussions. Technology-enhanced learning in sustainable design boosted motivation and engagement, contributing to students' attitudes toward sustainability. Connecting content with students' fields of study facilitated their participation in sustainability discussions ( Altomonte *et al.*, 2016; Gatti *et al.*, 2019; Hilty & Huber, 2018; Istenic Starcic *et al.*, 2018; Liu *et al.*, 2019).

In the professional training domain, online interactive databases provide access to educational content related to sustainability. Training for instructors in food nutrition, energy efficiency, and sustainable agriculture within Education for Sustainable Development (ESD) enhances sustainability literacy. Engagement with online platforms fostered sustainable behaviors and environmentally conscious actions, including innovation and supportiveness. Interactive digital stories proved valuable in engaging future teachers and improving their learning outcomes ( Delgado-Plaza *et al.*, 2021; Horng *et al.*, 2022; Kabadayi *et al.*, 2022; Shelton *et al.*, 2016).

Using Information and Communication Technology (ICT) tools to promote socio-ecological sustainability in community education for sustainable development (ESD) holds significant potential. These technology-enhanced learning systems can help people gain knowledge, improve critical thinking, change their attitudes, and take concrete actions for socio-ecological sustainability ( Aguayo & Eames, 2017).

The study, which included a mixed level of education, such as YouTube's role in informal learning, highlights its relevance and significance as a tool for education for sustainable development. ( Colás-Bravo & Quintero-Rodríguez, 2023). The study involving the Eco platform found that Eco significantly improved participants' environmental literacy and systems thinking. This enhanced understanding extends to complex ecosystem dynamics and contemporary environmental issues, such as biodiversity loss and overfishing ( Fjællingsdal & Klöckner, 2019). The study by Rodríguez-Loinaz *et al*. in 2022 supports the idea that educational initiatives can align science education and sustainable development, equipping students with global skills to address 21st-century sustainability challenges.

### 3.6 Word frequency among the selected articles

A word frequency analysis was conducted using NVIVO 12 software to identify the ten most frequently occurring terms within the selected articles, as presented in Table 2. Across all studies, the relevance and proximity of the topics to the target study groups emerged as critical factors strongly influencing the effectiveness of sustainability literacy enhancement through digital science communication.

**Table 2.** Word Frequency in the selected articles.

Word	Count	Weighted Percentage
learning	1644	1,23%
education	1230	0,92%
students	932	0,70%
sustainability	905	0,68%
environmental	818	0,61%
game	795	0,60%
sustainable	675	0,51%
study	543	0,41%
development	534	0,40%
research	516	0,39%

The word cloud visualization highlights key recurring terms such as “learning,” “education,” “students,” “sustainability,” “environmental,” and “game.” This reinforces the central role of digital tools in promoting sustainability literacy across various educational levels and contexts.

## 4. Discussion

This study conducted a scoping review to examine articles related to the role of digital science communication in enhancing sustainability literacy. It sought to answer five key research questions, including the academic settings and fields where these studies were conducted, the digital communication tools and approaches employed to shape learners' sustainability practices, the impacts of such communication tools on sustainability literacy, the obstacles and variables associated with its application, and the evidence supporting its contribution to achieving Sustainable Development Goals (SDGs) targets 4.7, 12.8 and 13.3 related to sustainability literacy.

In education, a comprehensive exploration of digital science communication's impact on sustainability learning is screened by various digital tools.

Extended Reality (XR) technologies, including Mixed Reality (MR), Virtual Reality (VR), and Augmented Reality (AR), have been integrated into educational settings at both primary and tertiary levels to deepen students' understanding of sustainability ( Aguayo & Eames, 2023; Liu *et al.*, 2019). In addition to XR technologies, various educational interventions such as serious games and simulation games have been employed to influence the sustainable behavior of students. These interactive and experiential learning methods are particularly effective in teaching complex concepts related to sustainability, as they allow students to experiment with different scenarios and see the immediate impact of their decisions at primary and tertiary education levels, demonstrating their versatility and effectiveness ( Fjællingsdal & Klöckner, 2019; Mercer *et al.*, 2017; Robin *et al.*, 2017; Veronica & Calvano, 2020). E-learning approaches have also played a significant role in modern education, particularly in secondary and tertiary education. Tools such as explanatory videos, WebQuests, and Digital Storytelling (DST) are used extensively through online portals and mobile applications. These methods provide flexible and accessible ways for students to learn at their own pace and convenience, enhancing their understanding of various subjects, including sustainability ( Altomonte *et al.*, 2016; Andriopoulou *et al.*, 2022; Delgado-Plaza *et al.*, 2021; Hilty & Huber, 2018; Horng *et al.*, 2022; Istenic Starcic *et al.*, 2018; Shelton *et al.*, 2016). Furthermore, methods like the Socio-Scientific Issues of Online Argumentation Patterns (SOAP) and social media platforms have been utilized to analyze the impact of educational interventions. These methods help educators understand how students engage with sustainability content and measure the effectiveness of these interventions in fostering positive attitudes towards sustainability ( Colás-Bravo & Quintero-Rodríguez, 2023; Fjællingsdal & Klöckner, 2019; Rodríguez-Loinaz *et al.*, 2022; Tsai, 2018).

Additionally, a case study in Chile examines digital science communication's role in community-based learning about sustainable development and environmental challenges in southern Chile, revealing that digital science communication effectively fostered profound and transformative education, locally rooted ecological literacy, active engagement, and the adoption of sustainable living principles and practices within certain community members ( Aguayo & Eames, 2017). Lastly, some studies employing mixed sample groups explore informal learning on platforms like YouTube, assess the effectiveness of serious games like Eco in raising awareness, and investigate how citizen science supported by ICT can enhance transformative teaching in sustainability education. However, several studies have indicated that obstacles, including a lack of familiarity with the tools, challenges related to tool design, and concerns about the feasibility and flexibility of digital tools, serve as prominent barriers ( Colás-Bravo & Quintero-Rodríguez, 2023; Fjællingsdal & Klöckner, 2019; Rodríguez-Loinaz *et al.*, 2022).

In summary, enhancing sustainability literacy through digital science communication can be distilled into three key points, as evidenced by the studies referenced in the range.

- Interactive Communication Capability: Both students/learners and professionals or community members benefit from the ability to engage in interactive communication facilitated by digital science communication. This dynamic engagement promotes a more profound understanding and participation in sustainability-related learning and initiatives.
- Contextualized Knowledge Availability: Digital tools deliver context-specific knowledge tailored to diverse communities and environments' unique needs and challenges. This ensures that sustainability literacy is relevant and applicable to real-world contexts.
- Flexible Learning Opportunities: Digital science communication offers flexible learning opportunities regarding timing and pace. Learners can access educational content conveniently, accommodating individual preferences. This flexibility enhances the accessibility and inclusivity of sustainability education.

By providing students with the ability to virtually explore distant locations or engage in experiments that may be logistically challenging in a traditional school setting, digital science communication transcends these barriers. It empowers learners to access transformative educational experiences that were previously inaccessible or impractical.

As evident from the scoping literature review, we can highlight eight points that can be facilitated by digital science communication to enhance sustainability literacy.

- Enhanced Understanding
- Awareness and Knowledge
- Attitude Change
- Virtual Accessibility
- Experiential Learning
- Community Engagement
- Data-Driven Learning
- Inclusivity and Proximity

## 5. Limitations

This scoping review provides a mapping of the literature on digital science communication in Education for Sustainable Development; however, several limitations should be noted. Firstly, while an initial broad search was conducted across Web of Science (WoS) and Scopus, the detailed eligibility assessment, filtering, and full-text screening were performed solely on the WoS dataset. This decision was based on WoS's enhanced functionality, including granular disciplinary categorization ("Education Educational Research") and the ability to filter records by specific Sustainable Development Goals (SDGs), which closely aligned with the thematic focus of this review and are not available in Scopus. Nevertheless, this selective approach may have excluded potentially relevant studies uniquely indexed in Scopus or other databases. Furthermore, a portion of the 309 retrieved records could not be accessed or retrieved, and thus were not screened, potentially limiting the comprehensiveness of the review.

Secondly, the literature included in this review is predominantly drawn from studies conducted in developed countries or the Global North, reflecting an evident geographical concentration. This imbalance limits the transferability of insights to educational and sustainability contexts in the Global South, where digital access, educational infrastructures, and socio-cultural dynamics may differ significantly. Addressing this gap represents a key area for future research. Additionally, although the review adhered to rigorous PRISMA-ScR methodological guidance, the screening, eligibility assessment, and data charting were conducted by a single reviewer, while procedures were applied consistently and transparently; this may introduce subjective bias in the inclusion of studies and thematic synthesis. Future reviews could strengthen reliability by using multiple independent reviewers and consensus mechanisms.

Finally, as a scoping review, the objective was to map and characterize the breadth of existing evidence rather than to assess the methodological quality of interventions. Therefore, while this review offers a productive overview, it does not provide a critical appraisal of study rigor or outcomes, which should be undertaken in subsequent systematic reviews or meta-analyses.

## 6. Conclusions

Based on the findings of this scoping literature review, the review suggests that pedagogical approaches enriched by digital science communication play a pivotal role and can meaningfully contribute to advancing Sustainable Development Goals (SDGs), particularly targets 4.7 (Quality Education), 12.8 (Responsible Consumption), and 13.3 (Climate Action). These targets are central to fostering sustainability literacy within the Education for Sustainable Development (ESD) framework. The review of the final selected studies published between 2016 and 2023 reveals diverse participant samples across various educational levels, with most research concentrating within the tertiary education domain. Geographically, most included studies originate from European countries, with some representation from Australia, Asia, South America, and North America, indicating a prevailing focus on developed nations or the Global North in the existing body of literature. Sample sizes varied, primarily encompassing the education sector, with limited involvement from tourism, business, and development fields. Notably, most of the identified articles are published in journals like "Sustainability" and the "International Journal of Sustainability in Higher Education." All selected articles included in the in-depth analysis were published only after the adoption of the SDGs in 2015, even though the review's timeframe started in 2000.

While the role of digital science communication in enhancing sustainability literacy within the ESD framework is well-established across the selected articles, this review highlights several critical gaps in the current research landscape that warrant future empirical investigation. A notable challenge lies in the limited availability of readily tailored online content, such as animation videos or games, specifically designed for sustainability literacy. Furthermore, many studies do not explicitly articulate the rationale behind their selection of specific digital science communication tools, approaches, or participant groups, limiting the transferability of their methods. Crucially, the long-term impacts of digital science communication interventions on sustainability literacy have not been sufficiently verified, making it difficult to ascertain which tools or approaches are most effective or should be avoided in fostering lasting sustainability behaviors. This is compounded by a scarcity of studies that include follow-up assessments or interviews with learners' families or teachers to track behavioral change over time.

In summary, integrating digital science communication into pedagogical practices is a powerful catalyst for advancing key SDG targets, underpinning sustainability literacy through interactive engagement, contextualized knowledge dissemination, and flexible learning opportunities. This review suggests that digital approaches can help overcome traditional constraints and enrich sustainability literacy across various educational settings. While some critics associate increased digital engagement with a disconnection from nature ( Fletcher, 2017), the findings here indicate that digital science communication can instead foster meaningful connections to the natural world and promote environmental awareness. These insights should be interpreted within the scope of literature predominantly sourced from Web of Science and reviewed by a single researcher, which may affect comprehensiveness and generalizability. Future research with longitudinal designs and more diverse regional representation is essential to deepen our understanding of long-term impacts and effectiveness.

## Ethics and consent

Ethical approval and consent were not required.

## Data Availability

No data associated with this article. Zenodo. Extended Data of Digital Science Communication for Sustainability Literacy: A Scoping Review Paper https://doi.org/10.5281/zenodo.16632684 ( Aung, 2025) The project contains the following extended data: Extended Data Table. Analysis Matrix and NVIVO12 data of reviewed articles Data are available under the terms of the Creative Commons Attribution 4.0 International license (CC-BY 4.0). Zenodo. Extended Data of Digital Science Communication for Sustainability Literacy: A Scoping Review Paper https://doi.org/10.5281/zenodo.16632684 ( Aung, 2025) The project contains the following reporting guidelines files: PRISMA-ScR Checklist PRISMA-ScR Flowchart Data are available under the terms of the Creative Commons Attribution 4.0 International license (CC-BY 4.0).
