# Digital science communication for sustainability literacy: A scoping review

**DOI:** 10.12688/openreseurope.20966.2

**Published:** 2026-06-24

**Authors:** Tin Shine Aung, Cláudia Faria, Joana Sousa, Mónica Mendes

**Affiliations:** 1 Ciências da Sustentabilidade, CIEBA, ITI-LARSyS, Universidade de Lisboa, Alameda da Universidade, 1649-004 Lisboa, Portugal; 2 UIDEF, Instituto de Educação, Universidade de Lisboa, Alameda da Universidade, 1649-013 Lisbon, Portugal; 3 Instituto de Saúde Ambiental, Faculdade de Medicina, Universidade de Lisboa, Av. Prof. Egas Moniz MB, 1649-028 Lisboa, Portugal; 4 ITI-LARSyS, Faculdade de Belas-Artes, Universidade de Lisboa, Largo da Academia Nacional de Belas-Artes, 1249-058 Lisboa, Portugal

**Keywords:** Behaviour Change, Digital Science Communication, Education for Sustainable Development (ESD), Information and Communication Technology (ICT), Sustainability Literacy

## Abstract

**Background:**

Global challenges such as climate change and resource depletion stem from unsustainable human activities, highlighting the critical need for widespread sustainability literacy to achieve the Sustainable Development Goals (SDGs). Education for Sustainable Development (ESD) is a key enabler, fostering holistic learning and action across all levels. Given this critical need, integrating digital science communication into ESD is essential.

**Objective:**

This scoping review explores how digital science communication contributes to sustainability literacy within ESD, focusing on advancing SDG targets 4.7, 12.8, and 13.3. Design Guided by the PRISMA-ScR framework, we systematically reviewed 20 peer-reviewed articles published between 2000 and 2023. An initial search was conducted across Scopus and Web of Science, with the final eligibility screening and analysis based on the Web of Science dataset, selected for its robust filtering capabilities within educational and sustainability contexts.

**Results:**

The findings show that immersive technologies (e.g., VR/AR), game-based learning, and online platforms are the most frequently used digital approaches. These tools support sustainability literacy by enabling interactive engagement, contextualised learning, and flexible access to educational content. However, their application is uneven across regions and educational levels. Key challenges include limited evidence of long-term behavioural change, restricted accessibility to advanced technologies, and insufficiently tailored content.

**Conclusion:**

Digital science communication can support sustainability literacy and help reconnect people with nature and deepen their understanding of sustainable practices. However, its effectiveness depends on pedagogical integration, accessibility, and stronger alignment with behaviour change frameworks. Further research is needed to assess long-term impacts and expand evidence across diverse geographical contexts.

## Plain language summary for Open Research Europe (ORE)

Climate change, pollution, and the overuse of natural resources are major global problems caused by how people live and use the planet’s resources. People everywhere should understand how to live sustainably to solve these challenges and achieve the United Nations Sustainable Development Goals (SDGs). This understanding is called sustainability literacy, which means having the knowledge, skills, and values to make thoughtful and responsible decisions supporting a healthy planet, fair societies, and strong communities. One way to build sustainability literacy is through Education for Sustainable Development (ESD), which helps people learn how to contribute to a better future. Today, digital tools like virtual reality, edutainment games, videos, and online learning platforms are used in schools, universities, and communities to support this learning. This review examined 20 research articles published between 2000 and 2023 to determine how well these digital tools help people understand sustainability. Using a method called scoping review and a software program called NVIVO12, we found that digital tools can make learning more interactive, flexible, and connected to real-world issues. These tools help learners understand complex sustainability topics better and encourage them to think and act differently. However, the review also showed that we need more research to know if these tools lead to long-term behavior change and to improve how these tools are designed and used. In short, digital science communication can play an important role in helping people understand and adopt sustainable practices.

## 1. Introduction

The world faces many formidable challenges, including the interconnected crises of food, finance, climate, and global recession, which have significant implications for sustainable development and environmental management ( UNDESA, 2022). These ongoing climate change crises, far-reaching consequences, and the exploitation of natural resources are intricately linked to human behaviors. Humans’ unsustainable actions have fundamentally altered the Earth’s ecosystem, directly threatening human lives. The evidence of dramatic and rapid global warming is indisputable, with two-thirds of the 1 °C increases in global temperatures occurring in just the past century, highlighting the human impact and responsibility ( UNESCO, 2020). According to the International Panel on Climate Change (IPCC), we must make rapid and comprehensive changes across all facets of society to mitigate the effects of global warming and limit it to 1.5 °C by the end of this century. This underscores the need to address environmental challenges and the broader spectrum of social and economic issues ( Allen *et al.*, 2018).

### 1.1 Education for sustainable development

Nearly 200 nations expanded the scope from the Millennium Development Goals (MDGs) to the Sustainable Development Goals (SDGs) at the 2015 United Nations Summit ( WHO, 2015). To effectively implement these goals globally, many of the world’s population should grasp the holistic concept of SDGs, recognize their accountability for their actions, and actively contribute to the vision of a sustainable future ( Glavič, 2020). Therefore, enhancing existing knowledge, values, and attitudes regarding sustainability among individuals is paramount, enabling them to make behavioral changes to support a sustainable future. The younger generation, in particular, should possess sufficient sustainability literacy to assume responsibility for their actions and actively participate in shaping a sustainable future. Education plays a pivotal role in realizing these sustainability objectives. Instilling a foundational understanding of sustainable development among youth is crucial for monitoring their evolving perceptions and behaviors related to consumption and sustainability literacy ( Chen *et al.*, 2022).

Education for Sustainable Development (ESD) represents a holistic and transformative educational approach, addressing content, learning outcomes, pedagogy, and the learning environment. This underscores the importance of equipping individuals with the knowledge, skills, values, and attitudes that empower them to actively engage in collaborative efforts for sustainable development initiatives ( Lozano *et al.*, 2017). Therefore, Education for Sustainable Development (ESD) is a lifelong learning process recognized as a crucial catalyst for achieving all 17 Sustainable Development Goals, notably contributing to Goal 4 on Education ( Smaniotto *et al.*, 2020). Individual sustainability literacy can be defined as the mindset and capabilities required to maintain sustainable actions and contribute to establishing a sustainable future ( Diamond & Irwin, 2013). Raising public awareness of sustainability and fostering the necessary skills for effective change is crucial in shaping consumers’ perceptions of their choices and enhancing their active engagement in research and development endeavors for scientific progress ( Katsaliaki & Mustafee, 2014).

Furthermore, ESD is important in three key SDGs: Goal 4 (Quality Education), Goal 12 (Responsible Consumption and Production), and Goal 13 (Climate Action). Aligned with Target 4.7: the objective for 2030 is to ensure that all learners acquire the knowledge and skills necessary to promote sustainable development. This includes fostering education for sustainable development and encouraging the adoption of sustainable lifestyles. Specifically, Target 12.8 highlights the importance of ensuring that individuals worldwide have access to relevant information and awareness about sustainable development, enabling them to make informed choices that align with environmental sustainability. Moreover, Target 13.3 underscores the need to enhance education, understanding, and institutional capacity to address climate change. This includes efforts in mitigation, adaptation, impact reduction, and early warning systems, reinforcing the role of education in building climate resilience. ( Diamond & Irwin, 2013; Katsaliaki & Mustafee, 2014; Lozano *et al.*, 2017; Smaniotto *et al.*, 2020).

### 1.2 Digital science communication

Information and Communication Technology (ICT) has effectively transformed our world into a global village driven by globalization. Within this evolving landscape, education, ICT, and innovation in science and technology have emerged as the three foundational pillars of the knowledge society ( Malik, 2018). The human brain benefits greatly from visual data representations to comprehend complex scenarios. Visual materials such as diagrams, schematics, drawings, and, more recently, photographs, films, and satellite images have been instrumental in conveying scientific discoveries since the early days of modern science ( Franconeri *et al.*, 2021). The well-known saying, “A picture is worth a thousand words,” underscores the comprehensive understanding that audiences can gain from visual materials ( Bucchi & Saracino, 2016; Siricharoen, 2013). In digital communication, knowledge generation and the dissemination of scientific information are becoming increasingly inclusive and socially oriented. To effectively engage with the non-scientific community, science communicators and educators employ the concept of “edutainment,” especially with the availability of new technologies in modern higher education institutions ( Anikina & Yakimenko, 2015).

In theory, outreach science communication encompasses knowledge sharing, raising awareness, and educating non-scientists, as seen in science journalism and citizen science initiatives ( Bonney *et al.*, 2016; Dickel & Franzen, 2016; Illingworth & Allen, 2020). While simplifying intricate scientific information to make it accessible to a lay audience is commendable, it is essential to strike a balance, as excessive simplification can lead to misconceptions about complex concepts and study limitations ( Kloepper, 2017). According to Fontaine *et al.* (2019), 70% of the public seeks scientific information on the Internet, and digitalization empowers scientists and individuals to communicate transparently about their research. A cross-media approach employing digital multimedia tools offers a powerful means of presenting the dynamic nature of scientific topics in engaging and understandable ways ( Rigutto, 2017). Multiple case studies in domains including healthy weight management, tobacco control, and vaccination uptake demonstrated that Digital Media for behavior change encompasses three primary methods: digital media interventions, formative research employing digital media, and digital media for conducting evaluations. Hence, digital (online visual) science communication involves transferring scientific knowledge through informative graphical content and animated videos delivered via educational multimedia platforms such as websites, mobile applications, edutainment games, broadcasting channels, and video communications, reaching a diverse audience ( Evans *et al.*, 2022).

### 1.3 Integration of information and communication technology in science education

For many years, educational courses were structured around textbooks, with teaching primarily delivered through lectures and presentations, complemented by tutorials and learning activities designed to reinforce and practice the subject matter ( Yusuf *et al.*, 2013). Traditional education paradigms have relinquished their exclusive control over the primary content of student learning. This content has transcended geographical boundaries and is now disseminated within the boundless realm of the internet ( Fauville *et al.*, 2013). Therefore, conventional educational institutions no longer hold exclusive control over knowledge, as we now exist and acquire knowledge within the virtual knowledge ecosystem. With this virtual knowledge ecosystem, information undergoes continuous amalgamation, enrichment, and evolution ( Breck, 2006). Traditional teaching underscored content delivery, while present-day educational contexts increasingly prioritize curricula fostering competence and enhanced performance. Present-day curricula strongly emphasize capabilities and the practical utilization of information and communication technology over mere theoretical knowledge of such technology ( Breck, 2006; Fauville *et al.*, 2013; Yusuf *et al.*, 2013).

Information and communication technology (ICT) is an integral component of today’s world, necessitating adjustments in culture and society to address the demands of the knowledge age. The adoption of ICT has brought about fundamental shifts in the practices and procedures across a broad spectrum of fields, encompassing economics, bureaucracy, social communication, and civil administration, including science education ( Nadda, 2022). Educational research in Japan, Canada, and Nigeria has revealed that heightened exposure to educational ICT integration within the curriculum positively affects student academic performance, notably in science-based subjects ( Laronde *et al.*, 2017). Hence, ICT assumes the role of a transformative agent within higher education. On the other hand, growing evidence of climate change, global warming, and financial and socio-cultural concerns are elevating the importance of sustainability within higher education curricula ( Leal Filho, 2000). The theme of ‘Digital Science Communication, ICT, and Education for Sustainable Development (ESD) constitutes a three-fold strategy, encompassing the instruction of sustainable development, the utilization of digital technologies, including ICT, and the behavior change through innovative and interactive pedagogical methods ( Ricard *et al.*, 2020).

### 1.4 Pandemic impact on sustainable development

The COVID-19 pandemic is one of this century’s deadliest biological threats to humanity, rivaling the 1918 Flu pandemic ( Khan *et al.*, 2020). Its impacts on human well-being and pursuing Sustainable Development Goals are profoundly distinct. Additionally, the pandemic introduces interference with the attainment of Sustainable Development Goals, which form the vital foundation for addressing global challenges such as hunger, inequality, natural resource conservation, and bolstering climate change adaptation for the long-term protection of our planet for future generations ( Hörisch, 2021). In response to COVID-19, the World Health Organization (WHO) and nearly all national governments implemented preventive measures like social distancing, lockdowns, and stay-at-home directives. These measures substantially impacted individuals’ daily routines and behaviors ( Marroquín *et al.*, 2020).

Consequently, numerous researchers explored the multifaceted impacts of COVID-19 on various aspects of life. These impacts on sustainability were immense and posed significant challenges to the global pursuit of Sustainable Development Goals. They encompassed the closure of schools, effects on mental well-being, travel restrictions, alterations in work patterns, labor shortages, home isolation, and many more impacts ( Azevedo *et al.*, 2021). According to the United Nations, school closures affected 91% of students worldwide, and long-term home isolation heightened the risk of mental health issues among students ( Lee, 2020).

Paradoxically, the pandemic also yielded unexpected positive outcomes concerning Sustainable Development Goals. For instance, technology played a pivotal role in bridging gaps in the education system in Ireland, enabling continuous education for students through digital communication channels, thereby contributing to the realization of quality education as per SDG 4 ( Mohan *et al.*, 2020). The usefulness of digital tools is becoming widespread, affecting daily areas of life, including social, economic, and political aspects. The COVID-19 pandemic has made having a digital presence even more critical ( Davis *et al.*, 2014; Sá *et al.*, 2022). Behavioral change can be defined as the sustained alteration of individual habits and lifestyles ( Alturki & Aldraiweesh, 2021). WHO and various national governments engaged in health education and promoted behavioral adaptations to the pandemic through digital science communication using multimedia.

During the pandemic, the use of digital communication channels has also supported progress towards SDG 3 (Good Health and Well-being), particularly by overcoming the physical absence of instructors and enabling behaviour change through distance learning. Studies by ( Pedersen *et al.*, 2021 and Wang & Huang, 2021) show that these approaches can help bridge geographical gaps and improve health-related outcomes during the COVID-19. From a theoretical perspective, these developments can be understood through constructivist and transformative learning approaches within Education for Sustainable Development (ESD). These approaches emphasise active and experience-based learning, where individuals reflect on their values and develop new perspectives. This is especially important in education for sustainability, where learning is expected to influence attitudes and behaviour. In addition, behaviour change theories suggest that long-term change depends on continued engagement and learning that is relevant to real-life contexts ( Schunk, 2020; Steg & Vlek, 2009; Taylor & Cranton, 2012). However, these theoretical perspectives are not always clearly applied or discussed in studies on digital science communication and sustainability literacy.

### 1.5 Purpose of the study

Despite the increasing use of digital tools in education for sustainability, existing research remains fragmented across disciplines, educational levels, and geographical regions. There is limited synthesis of which tools are more effective, how their impacts differ, and what challenges affect their implementation. This highlights the need for a structured review to map current evidence and identify gaps in the field.

Therefore, this scoping review examines how digital science communication enhances learners’ sustainability literacy within the broader Education for Sustainable Development (ESD) framework and the 2030 Agenda. The review explores the impacts and obstacles associated with using digital tools to foster sustainability-related knowledge, values, and behaviors among learners across diverse educational and geographical settings. Sustainability literacy refers to the knowledge, skills, and values that empower individuals to make informed decisions and take responsible actions that advance environmental protection, economic viability, and social equity. These competencies are considered critical for achieving the Sustainable Development Goals (SDGs), particularly targets 4.7, 12.8, and 13.3 ( UNESCO, 2020). The SDGs are recognized as a universal framework for promoting peace and prosperity for humanity and the planet, both now and in the future ( Agbedahin, 2019; De Jong & Vijge, 2021; Griffiths, 2020).

This study adopts a scoping review approach to systematically map existing research across different levels of education (Population), focusing on digital science communication tools and practices (Concept), within the context of Education for Sustainable Development (Context).

The review is guided by three main objectives:

▪ To examine the educational contexts, including levels, fields, and geographical distribution, in which digital science communication has been applied;
▪ To identify the digital tools and approaches used to promote sustainability literacy; and
▪ To analyse their impacts, associated challenges, and contributions to sustainability education, while highlighting key research gaps and implications for future studies and policy development

## 2. Materials and methods

Given the exploratory nature of the topic, the diversity of digital communication tools, and the wide range of educational contexts in which they are applied, a scoping review approach was deemed appropriate to systematically map the extent, range, and characteristics of existing research. This methodology is well-suited for identifying knowledge gaps, clarifying conceptual boundaries, and informing future empirical studies in an emerging and fragmented field lacking prior comprehensive synthesis.

Accordingly, this review focuses on peer-reviewed scientific articles published between 2000 and 2023 that examine digital science communication or information and communication technologies (ICT) in Education for Sustainable Development (ESD), particularly on sustainability literacy. The post-2000 period was chosen to align with adopting the Millennium Development Goals (MDGs), which marked a global shift toward integrated development frameworks.

The research team discussed and drafted the study protocol by PRISMA-ScR guidelines; however, a formal review protocol was not registered or published beforehand. Despite this, the scoping review was conducted with strict adherence to the PRISMA Extension for Scoping Reviews (PRISMA-ScR) guidelines, thereby ensuring transparency, methodological rigor, and reproducibility at all stages of the review process ( Tricco *et al.*, 2018; Zimmerman *et al.*, 2023).

The study applied principles of qualitative data analysis and synthesis to achieve its aims, drawing on established techniques for qualitative evidence synthesis to identify and characterize the most relevant and representative studies in the field ( Sandelowski & Barroso, 2007).

### 2.1 Formulate *guiding research q*uestion

This scoping review aims to examine the role of digital science communication in enhancing sustainability literacy within the context of Education for Sustainable Development (ESD). To achieve this, the study addresses the following three research questions:

*RQ1: Contexts and applications*

In which educational contexts (e.g., academic levels and fields of study) and geographical locations has digital science communication been applied, and which digital tools and approaches are most commonly used to support sustainability literacy?

*RQ2: Impacts on sustainability literacy and behaviour*

How does digital science communication influence learners’ sustainability literacy, including their knowledge, attitudes, and behaviours related to sustainable development?

*RQ3: Challenges and contributions to SDGs*

What obstacles and influencing factors are associated with the application of digital science communication, and what evidence exists regarding its contribution to achieving Sustainable Development Goals (SDG) targets 4.7, 12.8, and 13.3?

### 2.2 Comprehensive initial search

**Figure 1.**
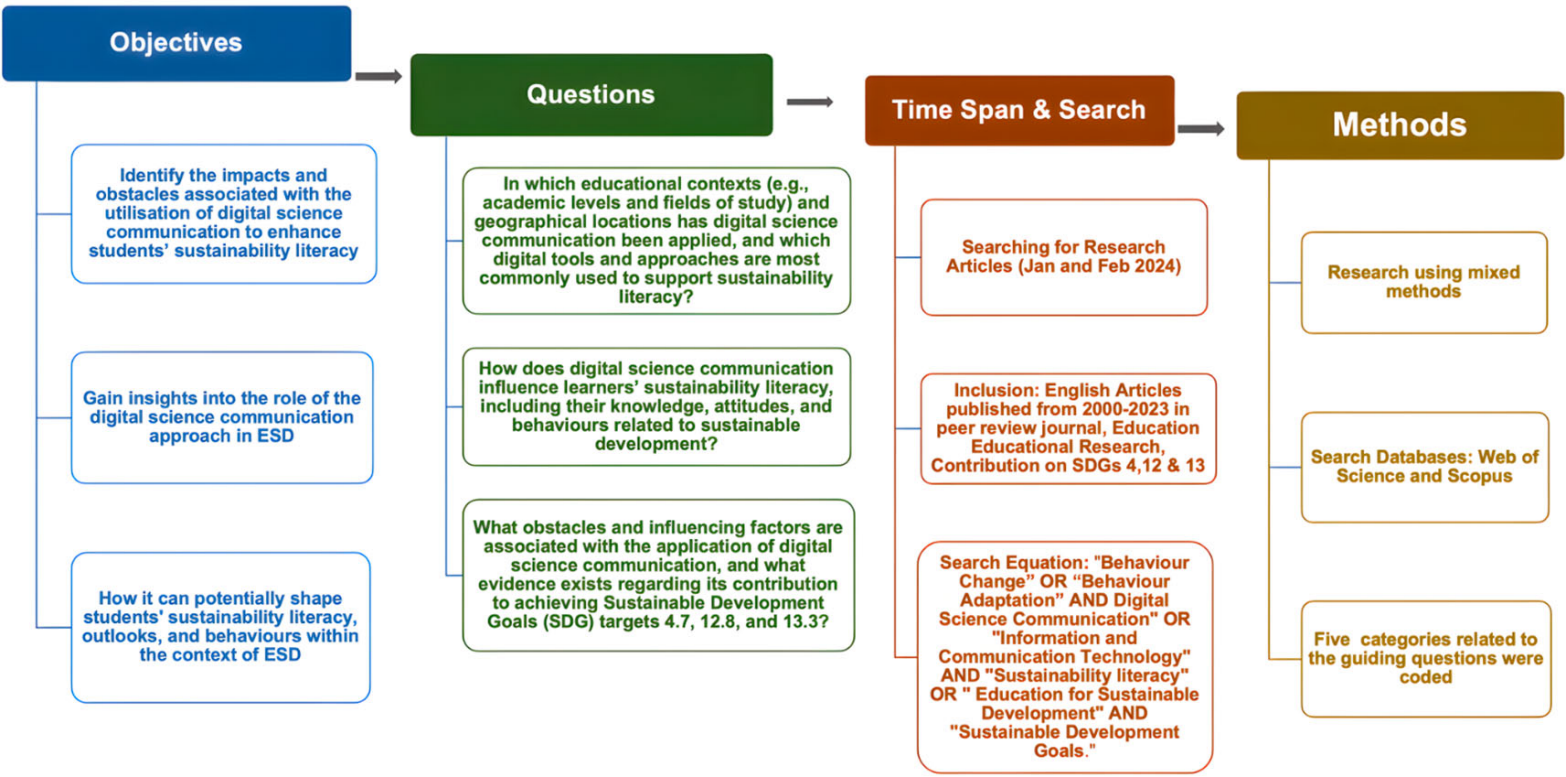
Conceptual framework and scope of the scoping review ( González-Salamanca *et al*., 2020).

A comprehensive initial search was conducted systematically and sequentially, emphasizing the broad retrieval of relevant articles to establish parameters related to topics, populations, timelines, and research methods based on ( González-Salamanca *et al*., 2020) ( Figure 1). This approach aligns with the principles for robust evidence synthesis as guided by the PRISMA Extension for Scoping Reviews (PRISMA-ScR), ensuring methodological rigor and transparency ( Tricco *et al.*, 2018).

**Figure 2.**
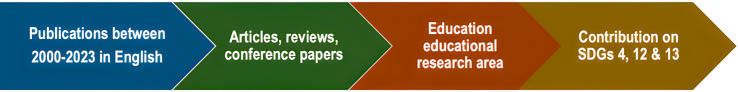
**Inclusion criteria** in the review process ( González-Salamanca *et al*., 2020).

**Table 1.** Distribution of selected studies across analytical categories and corresponding references.

Categories	Numbers	References
Academic Settings, Research Fields, and Locations	20	Aguayo & Eames; 2017; Aguayo & Eames, 2023; Altomonte *et al.*, 2016; Andriopoulou *et al.*, 2022; Colás-Bravo & Quintero-Rodríguez, 2023; Delgado-Plaza *et al.*, 2021; Fjællingsdal & Klöckner, 2019; Gatti *et al.*, 2019; Hilty & Huber, 2018; Horng *et al.*, 2022; Istenic Starcic *et al.*, 2018; Kabadayi *et al.*, 2022; Liu *et al.*, 2019; Martínez-Borreguero *et al.*, 2020; Mercer *et al.*, 2017; Robin *et al*., 2017; Rodríguez-Loinaz *et al.*, 2022; Shelton *et al.*, 2016; Tsai, 2018; Veronica & Calvano, 2020
Digital Science Communication Tools and Approaches	20	Aguayo & Eames; 2017; Aguayo & Eames, 2023; Altomonte *et al.*, 2016; Andriopoulou *et al.*, 2022; Colás-Bravo & Quintero-Rodríguez, 2023; Delgado-Plaza *et al.*, 2021; Fjællingsdal & Klöckner, 2019; Gatti *et al.*, 2019; Hilty & Huber, 2018; Horng *et al.*, 2022; Istenic Starcic *et al.*, 2018; Kabadayi *et al.*, 2022; Liu *et al.*, 2019; Martínez-Borreguero *et al.*, 2020; Mercer *et al.*, 2017; Robin *et al*., 2017; Rodríguez-Loinaz *et al.*, 2022; Shelton *et al.*, 2016; Tsai, 2018; Veronica & Calvano, 2020
Impacts of Digital Science Communication	20	Aguayo & Eames; 2017; Aguayo & Eames, 2023; Altomonte *et al.*, 2016; Andriopoulou *et al.*, 2022; Colás-Bravo & Quintero-Rodríguez, 2023; Delgado-Plaza *et al.*, 2021; Fjællingsdal & Klöckner, 2019; Gatti *et al.*, 2019; Hilty & Huber, 2018; Horng *et al.*, 2022; Istenic Starcic *et al.*, 2018; Kabadayi *et al.*, 2022; Liu *et al.*, 2019; Martínez-Borreguero *et al.*, 2020; Mercer *et al.*, 2017; Robin *et al*., 2017; Rodríguez-Loinaz *et al.*, 2022; Shelton *et al.*, 2016; Tsai, 2018; Veronica & Calvano, 2020
Obstacles and Variables Associated with the Application	9	Aguayo & Eames; 2017; Aguayo & Eames, 2023; Altomonte *et al.*, 2016; Colás-Bravo & Quintero-Rodríguez, 2023; Delgado-Plaza *et al.*, 2021; Fjællingsdal & Klöckner, 2019; Hilty & Huber, 2018; Martínez-Borreguero *et al.*, 2020; Shelton *et al.*, 2016
Evidence on Achieving Designated SDGs Targets for ESD	20	Aguayo & Eames; 2017; Aguayo & Eames, 2023; Altomonte *et al.*, 2016; Andriopoulou *et al.*, 2022; Colás-Bravo & Quintero-Rodríguez, 2023; Fauville *et al.*, 2013; Franconeri *et al.*, 2021; Griffiths, 2020; Hilty & Huber, 2018; Illingworth & Allen, 2020; Istenic Starcic *et al.*, 2018; Leal Filho, 2000; Marroquín *et al.*, 2020; Martínez-Borreguero *et al.*, 2020; Rigutto, 2017; Robin *et al.*, 2017; Rodríguez-Loinaz *et al.*, 2022; Shelton *et al.*, 2016; Tsai, 2018; Veronica & Calvano, 2020

The information-gathering process focused on selected databases, namely the Web of Science (WOS) and Scopus. The article search and selection period spanned from January to February 2024 and was conducted using the Virtual Private Network (VPN) of the University of Lisbon. Subsequently, inclusion criteria were defined based on ( González-Salamanca *et al*., 2020), as described in Figure 2. The review included peer-reviewed journal and conference articles published in English, as well as one peer-reviewed conference paper with full-text availability, because it met the inclusion criteria and was directly relevant to the research questions. During screening, studies were included only if they explicitly addressed digital science communication or ICTs in the context of education for sustainability or sustainability literacy in the scope of the study.

A search strategy employing a combination of specific keywords and Boolean operators (e.g., AND, OR), customized to the databases’ requirements. The complete and reproducible search strings used for each database are provided in the extended data. The search query included terms such as “Behaviour Change” OR “Behaviour Adaptation,” AND “Digital Science Communication,” OR “Information and Communication Technology”, AND “Sustainability literacy,” OR “Education for Sustainable Development”, AND “Sustainable Development Goals” in either the title or in the keywords. The time frame of 2000 to 2023 was selected to align with the launch of the Sustainable Development Goals (SDGs), which succeeded the Millennium Development Goals (MDGs), and to correspond with the global push for Education for Sustainable Development since then.

### 2.3 Initial study selection

**Figure 3.**
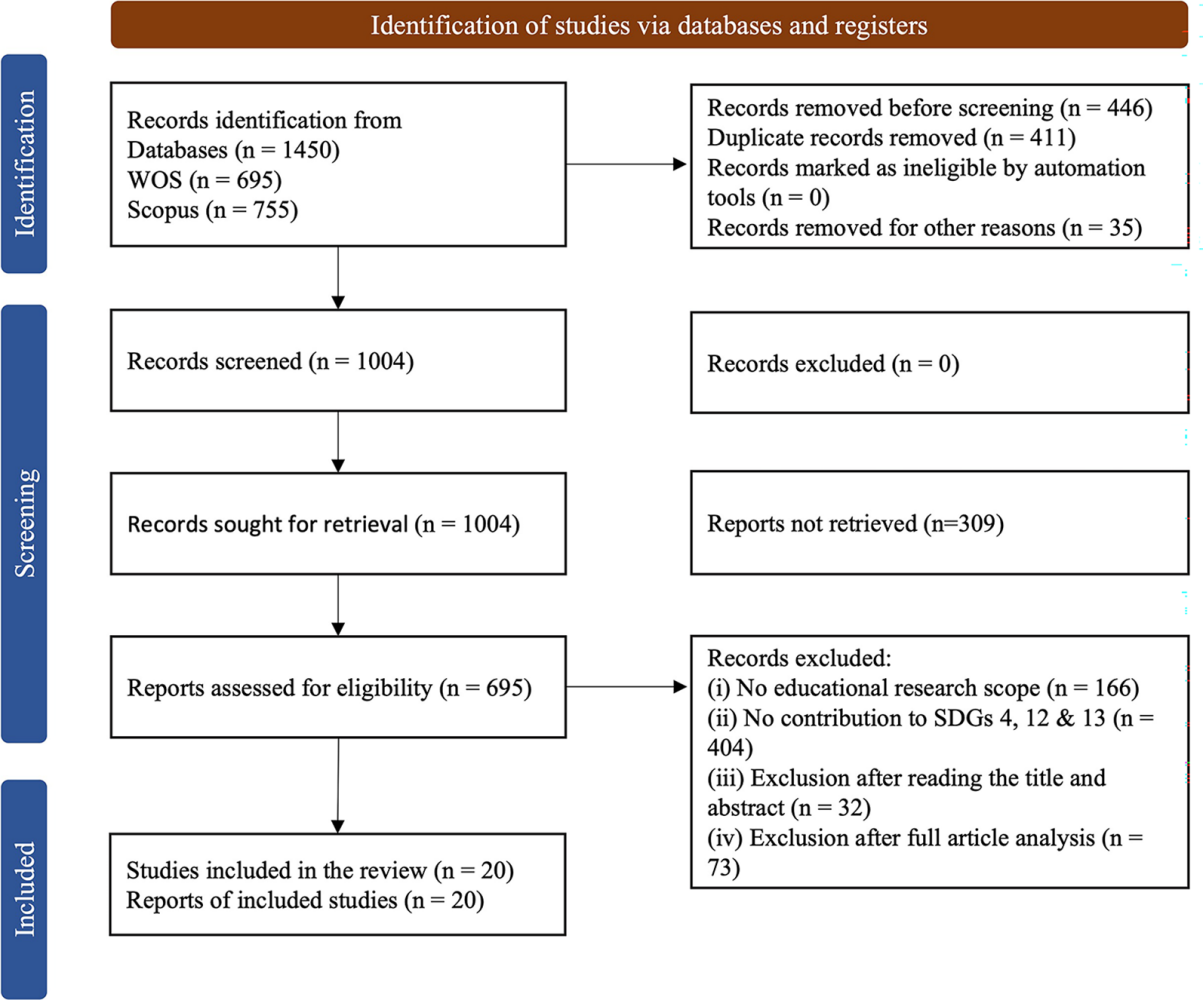
Schematic representation of the review process (Adhering to the PRISMA Protocol PRISMA-ScR ( Tricco *et al.*, 2018).

This scoping review was conducted by the PRISMA Extension for Scoping Reviews (PRISMA-ScR) guidelines ( Tricco *et al.*, 2018) to ensure methodological rigor, transparency, and reproducibility. A visual summary of the search and selection process is provided in Figure 3. Initial comprehensive searches were carried out using two major bibliographic databases: Web of Science (WoS) and Scopus, with the final search completed in February 2024. The full search strategies, including Boolean logic and keyword strings, are detailed in Section 2.2. The search yielded a total of 1,450 records, comprising 695 from WoS and 755 from Scopus. After exporting all records, a preliminary pre-screening process excluded 446 entries. This included 411 duplicates, identified and removed using EndNote, and 35 non-eligible records, such as conference abstracts that did not meet the inclusion criteria for full publication. This initial exclusion process primarily affected records from Scopus or duplicate entries where the Scopus version was discarded, ensuring the full 695 WoS records remained in the deduplicated dataset.

This resulted in 1,004 unique records entering the formal screening phase. Of these 1,004 unique records, the entire set of 695 records identified initially from Web of Science remained intact after the preliminary pre-screening. Upon further comparison, it became evident that the Web of Science (WoS) database offered two key methodological advantages that aligned closely with the aims and thematic focus of this review: (1) WoS provides a more granular subject classification, including a distinct disciplinary category for ‘Education Educational Research,’ which lacks a direct equivalent in Scopus; and (2) WoS supports direct filtering by specific Sustainable Development Goals (SDGs), namely, SDG 4 (Quality Education), SDG 12 (Responsible Consumption and Production), and SDG 13 (Climate Action), a functionality not currently available in Scopus. These features enabled a more precise and consistent application of the eligibility criteria in line with the objectives of this review. Accordingly, while both databases contributed to the initial mapping of the literature landscape, the primary screening and selection process was conducted exclusively on the WoS records. However, restricting the detailed screening to WoS may have excluded relevant studies indexed exclusively in Scopus.

The 695 unique WoS records (a subset of the 1,004 total unique records) were further refined using the platform’s automated filtering tools. Applying the disciplinary filter for ‘Education Educational Research’ reduced this dataset to 529 records. These were then further filtered to retain only those indexed under SDGs 4, 12, or 13, resulting in 125 records eligible for subsequent manual screening of abstracts, and eventual full-text review and thematic synthesis. The filtering process was conducted by reviewer Aung, T. S., following predefined inclusion criteria aligned with the conceptual focus on sustainability competence, with particular emphasis on sustainability literacy and adopting sustainable lifestyles.

### 2.4 Final selection and data charting

Titles and abstracts of the 125 records were initially screened, resulting in 93 articles retained for full-text review. A subsequent full-text assessment led to the exclusion of 73 articles that did not sufficiently align with the research questions or lacked clear contributions to the dimensions of sustainability competence. This process yielded a final sample of 20 articles selected for in-depth analysis.

The detailed review involved systematically extracting evidence relevant to the guiding research questions. To support this, an analysis matrix was developed encompassing the following elements:

1. Publication year
2. Bibliographic details
3. Academic settings, research fields, and geographical locations
4. Main themes and ideas addressed
5. Connections between the main themes and research questions
6. Textual segments containing relevant evidence
7. Indications of contributions to SDG targets 4.7, 12.8, and 13.3 relating to sustainability literacy

The complete analysis matrix was deposited in the Zenodo platform. (See extended data). The data from the 20 included studies were charted using a standardized extraction matrix developed in Microsoft Excel, which was pilot-tested on a sample of three articles to ensure clarity and consistency of categories. Data charting was conducted independently by the first reviewer (Aung, T. S.). To reduce potential bias associated with single-reviewer screening, predefined inclusion criteria were strictly applied throughout the selection process. All extracted data were systematically recorded using the structured matrix, and entries were reviewed to ensure consistency across predefined categories. Qualitative data were then imported into NVivo 12, where they were coded using five thematic categories aligned with the research questions. An inductive thematic approach was applied to organize and refine the codes. No authors of primary studies were contacted for additional data, as the full texts provided sufficient detail for extraction and synthesis.

### 2.5 Summary of findings from selected studies using qualitative techniques

The data was analyzed using NVIVO12 software. Based on the analysis matrix, the information was organized into five categories, each corresponding directly to one of the study’s guide questions. The coding process in NVIVO12 followed an inductive thematic approach. Initial codes were generated based on recurring concepts identified during full-text reading. These codes were subsequently grouped into broader categories aligned with the research questions. Each category was defined operationally before final analysis to ensure consistency and transparency in the coding process.

• Academic settings, research fields, and locations: Gather information regarding the educational level, field of study, and geographic locations where the studies were conducted.
• Digital science communication tools and approaches: Identify the various tools utilized to enhance learners’ sustainability literacy and how these tools influence changes in learners’ behaviors to promote greater sustainability.
• Impacts of digital science communication: Compiles the observed impacts resulting from the application of digital science communication in improving learners’ sustainability literacy.
• Obstacles and variables associated with the application: Recognises and examines the challenges and variables associated with implementing digital science communication approaches to enhance sustainability literacy among learners.
• Evidence on achieving designated SDG targets: Collects the available evidence demonstrating the contribution of digital science communication to attaining SDG targets 4.7, 12.8, and 13.3.

These classifications were employed during the analysis and interpretation phase, resulting in a distribution summarised in Table 1. This classification supports a structured comparison of how digital science communication has been examined in relation to sustainability literacy, including its applications, impacts, and associated challenges.

## 3. Results

The results are organised into five analytical categories derived from the data extraction and coding process. These categories collectively contribute to addressing the three research questions by examining:

(1) the contexts and applications of digital science communication ( Section 3.1 and 3.2),
(2) its impacts on sustainability literacy ( Section 3.3), and
(3) the associated challenges and contributions to Sustainable Development Goals (SDGs) ( Section 3.4 and Section 3.5).

In addition, a word frequency analysis ( Section 3.6) provides further insight into dominant themes across the selected studies.

### 3.1 Academic settings, research fields, and locations

In detail, four studies were conducted at the primary level, encompassing 30 participants in New Zealand, 50 pupils in Italy, 60 in the UK, and 6 participants in France ( Aguayo & Eames, 2023; Mercer *et al.*, 2017; Robin *et al.*, 2017; Veronica & Calvano, 2020).

Additionally, three studies were carried out at the secondary level, involving 81 participants in Spain, 153 participants in Greece, and 68 participants in Taiwan ( Andriopoulou *et al.*, 2022; Martínez-Borreguero *et al.*, 2020; Tsai, 2018).

Furthermore, seven studies were conducted at the tertiary education level, with 360 participants in China, 847 participants in Slovenia, 32 and 405 participants in the UK, 127 and 567 participants in Taiwan, and 80 and 54 participants in business schools of the university in Switzerland, respectively ( Altomonte *et al.*, 2016; Gatti *et al.*, 2019; Hilty & Huber, 2018; Horng *et al.*, 2022; Istenic Starcic *et al.*, 2018; Liu *et al.*, 2019; Mercer *et al.*, 2017).

Three studies focused on the professional training domain, with 143 participants in the Czech Republic, 102 in Ecuador, and 223 in the US ( Delgado-Plaza *et al.*, 2021; Kabadayi *et al.*, 2022; Shelton *et al.*, 2016).

Moreover, one study was conducted at the community level, involving 24 local community members from the target community in Chile ( Aguayo & Eames, 2017).

Lastly, four studies involved mixed levels of education, comprising 504 participants in Spain, 56 in Norway, and 127 and 68 in Taiwan, 221 in Greece, the UK, and Spain combined ( Colás-Bravo & Quintero-Rodríguez, 2023; Fjællingsdal & Klöckner, 2019; Rodríguez-Loinaz *et al.*, 2022; Tsai, 2018).

**Figure 4.**
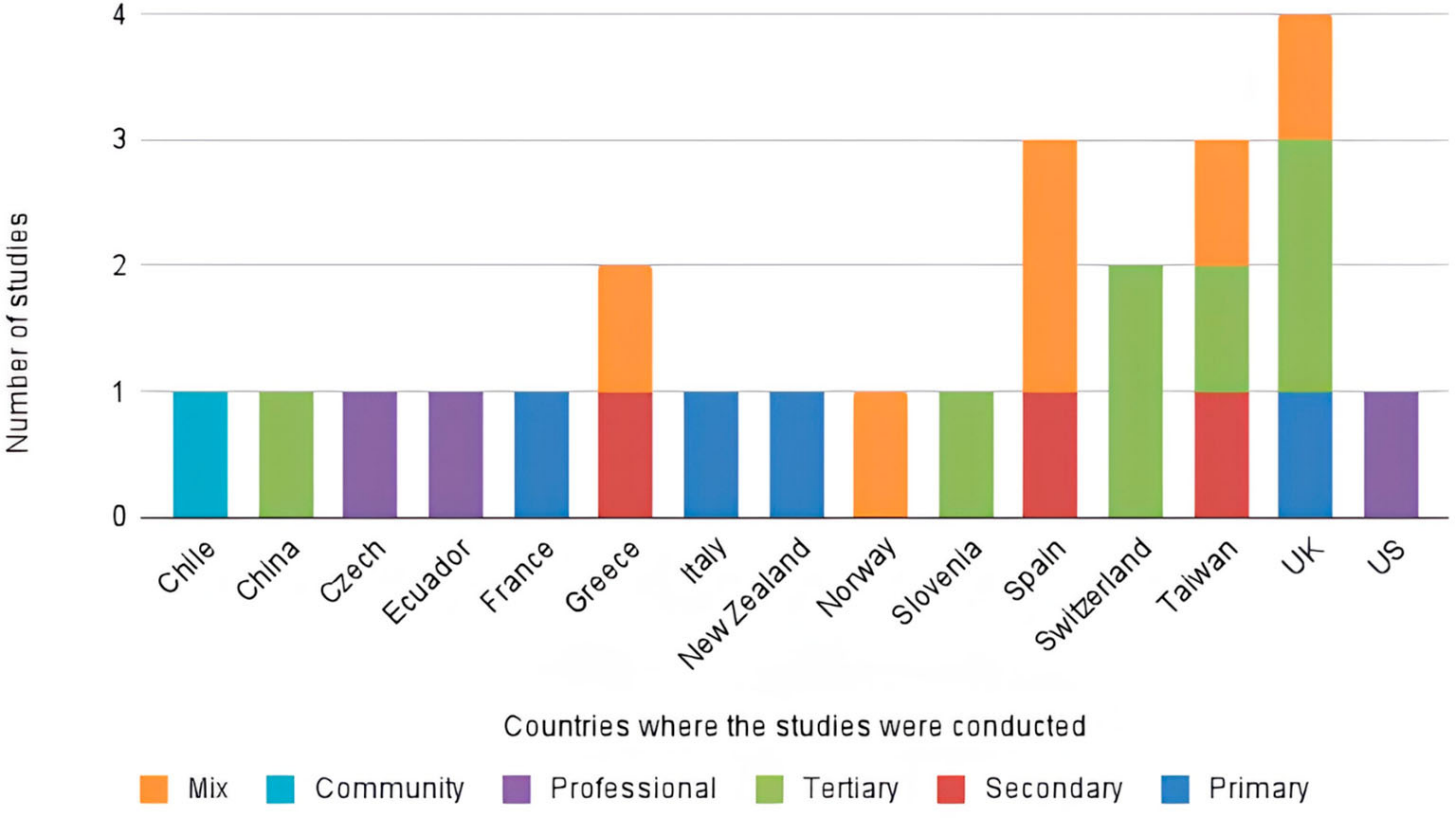
Selected Digital Science Communications Studies Conducted in Different Countries and Corresponding Levels.

Based on the data analysis, numerous studies have concentrated on utilizing digital science communication to improve sustainability literacy, with a notable concentration in European countries. A smaller number of studies were conducted in Australia, Asia, South America, and North America, particularly since the adoption of the UN SDGs in 2015. Moreover, there were significant variations in sample sizes among the studies. Most study samples were from education, although a few studies involved tourism, business, and development representatives. To better understand patterns in research production, the origin of the first author (assumed to represent the principal researcher) was compared with the geographical location of the study sample. The analysis shows that, in most cases, the study location corresponds to the country of the first author, indicating that research is generally conducted within national contexts. Only three exceptions were identified. A researcher from New Zealand conducted a community-level study in Chile ( Aguayo & Eames, 2017), and a researcher from Turkey carried out a professional-level study in the Czech Republic ( Kabadayi *et al*., 2022). In addition, one study involving mixed levels of education, led by a researcher from Spain, included participants from multiple countries, namely Greece, the UK, and Spain ( Rodríguez-Loinaz *et al*., 2022). Overall, these findings suggest limited cross-national research collaboration and a strong concentration of studies within Europe with limited representation from low- and middle-income countries. This may partly explain the geographical imbalance observed in the dataset. Notably, all selected studies were published after the adoption of the Sustainable Development Goals (SDGs) in 2015, despite the review covering the period from 2000 onwards ( Figure 4).

### 3.2 Digital science communication tools and approaches

In the primary education domain, these studies explored students’ experiences transitioning from virtual reality (VR) to natural marine environments, focusing on the educational impacts of immersive technologies like VR, augmented reality (AR), extended reality (XR), and 3D visualization ( Aguayo & Eames, 2023). Another study introduced educational interventions, including a serious game and an explanatory video, aiming to address the critical issue of marine waste and its impact on marine species. These initiatives aimed to improve students’ understanding of marine ecosystems, raise awareness about the environmental consequences of marine pollution, and promote the use of a specialised website for community-driven Education for Sustainable Development (ESD) projects ( Mercer *et al.*, 2017; Veronica & Calvano, 2020). This educational approach involves ongoing learning and problem-solving ( Robin *et al.*, 2017).

Three distinct digital science communication approaches were employed in the secondary education domain. The first study utilized resources like WebQuests and video games within a discovery learning model to investigate how these ICT-based interventions impact secondary school students’ sustainability content, encompassing cognitive and affective aspects ( Martínez-Borreguero *et al.*, 2020). The second study focused on Digital Storytelling (DST) as an instructional approach in a non-formal learning setting, specifically at the Hellenic Centre for Marine Research. It aimed to explore the effectiveness of DST in shaping positive attitudes among high school students toward topics related to plastic pollution and marine trash in the Mediterranean ( Andriopoulou *et al.*, 2022). Lastly, another study introduced the Socio-Scientific Issues of Online Argumentation Pattern (SOAP) as a pedagogical approach, empowering students to engage effectively in online arguments about Socio-Scientific Issues (SSI) ( Tsai, 2018).

In the scope of tertiary education, several studies tested different aspects of digital science communication tools for sustainability literacy. One study aimed to enhance environmental ethics education using virtual reality techniques. Another focused on students’ perceptions through their social impact on sustainable development awareness and behaviors. Additionally, a study explored the potential of interactive e-learning and m-learning courses in environmental disciplines to promote sustainable development education. Another study identified specific course content that engages students in sustainable development, particularly those in ICT-related programs. Lastly, a Swiss business school study investigated experiential learning through simulation games to teach sustainability concepts to business-specialized students ( Altomonte *et al.*, 2016; Gatti *et al.*, 2019; Hilty & Huber, 2018; Istenic Starcic *et al.*, 2018; Liu *et al.*, 2019). Additionally, a study with university students in tourism examined how using sharing economy platforms affected their learning and attitudes toward sustainability ( Horng *et al.*, 2022) and another study with university students to engage in online arguments about Socio-Scientific Issues (SSI) ( Tsai, 2018).

In the professional training domain, various studies focused on professionals like kindergarten principals and pre-service teachers. For instance, one study examined how kindergarten principals use technology, specifically digital science communication, to support sustainability-focused learning ( Kabadayi *et al.*, 2022). Another study explored online platforms and digital tools for long-term sustainable development after the COVID-19 pandemic ( Delgado-Plaza *et al.*, 2021). Lastly, a study with pre-service teachers used digital storytelling to enhance their understanding of sustainability science in online courses ( Shelton *et al.*, 2016).

Another case study explores how digital science communication is used for community-based learning about sustainable development in southern Chile. It investigates the environmental problems related to the degradation of a lake and the broader socio-ecological impacts caused by this issue ( Aguayo & Eames, 2017).

Some studies adopted a mixed model of sample groups. One study used quantitative methods to explore informal learning on YouTube for sustainable development programs, looking at age, gender, and education level as factors. They used statistical techniques to analyze the differences in informal learning ( Colás-Bravo & Quintero-Rodríguez, 2023). Another study aimed to assess the effectiveness of the online Eco platform by the US Department of Education in raising awareness about ecosystems ( Fjællingsdal & Klöckner, 2019). The last study, which also used a mixed sample of groups, explored how ICT-supported citizen science can enhance transformative teaching in education for sustainable development ( Fletcher, 2017).

A comparative analysis shows that immersive technologies and game-based approaches are among the most frequently used tools, followed by online learning platforms and digital storytelling. Other approaches, such as citizen science and argumentation-based models, appear less frequently, indicating emerging but less established practices.

### 3.3 Impacts of digital science communication

In the primary education scope, the first study discovered that most students enjoyed learning through virtual reality (VR) and recognized its potential for innovative education, especially when it included engaging elements like missions. It also showed that extended reality (XR) technologies could significantly improve students’ understanding of marine ecology, as evidenced by better post-test results. Furthermore, the research highlighted how augmented reality (AR) could connect students’ learning experiences across different contexts, emphasizing the role of digital science communication in enhancing sustainability literacy ( Aguayo & Eames, 2023). In the second study, a serious game aimed to improve ocean literacy, focusing on the biodiversity of the Apulian and Mediterranean seas to raise children’s awareness of marine life preservation. The study confirmed that children could acquire knowledge effectively through new technologies and serious games ( Veronica & Calvano, 2020). The third study enhanced students’ understanding of sustainability. It equipped them with problem-solving and design-thinking skills, fostering social responsibility and sustainable attitudes that could address real-life issues beyond the classroom. Understanding the unique characteristics and needs of the local community was crucial for providing impactful and culturally relevant learning experiences ( Mercer *et al.*, 2017).

In secondary education, the first study evaluated the impact of video games and WebQuests on students, focusing on their knowledge and attitudes toward sustainability. The results showed that these ICT educational activities effectively promoted learning, particularly among students who engaged more with video games ( Martínez-Borreguero *et al.*, 2020). The second study, a pioneering case, used digital storytelling to address plastic marine pollution, significantly improving students’ performance. This approach raised awareness and transformed attitudes towards marine litter issues ( Andriopoulou *et al.*, 2022). In the third study, the Socio-Scientific Issues of Online Argumentation Pattern (SOAP) facilitated interaction among educators and students, enhancing student engagement and participation ( Tsai, 2018).

Research in the tertiary education domain indicates that virtual reality experiences and practical courses can generate interest, improve the learning experience, and promote environmental ethics and practical skills. These experiences enhance cognitive learning and cultivate a strong sense of ecological responsibility, allowing participants to apply their knowledge and skills to real-world environmental challenges ( Liu *et al.*, 2019). Additionally, online social media plays a significant role in higher education for sustainable development, fostering connections and resources that support sustainability efforts. Integrating educational technology into higher education is vital, especially when teaching systems thinking in merging ICT and sustainable development disciplines ( Altomonte *et al.*, 2016; Hilty & Huber, 2018; Istenic Starcic *et al.*, 2018). A study by a university business school shows that students understand the importance of sustainability across an enterprise, making business simulations a valuable tool for sustainability literacy ( Gatti *et al.*, 2019). In the tourism sector, university students showed increased awareness of sustainability-related topics like environmental concerns and energy efficiency when using online platforms ( Horng *et al.*, 2022) and and university students engaged and participated in the Socio-Scientific Issues of Online Argumentation Pattern (SOAP) ( Tsai, 2018).

The professional training domain involving kindergarten principals and educators highlights the importance of sustainable learning practices for children, emphasizing responsible resource use in areas like materials, food, energy, and water. These studies indicate that ICT has substantial potential to enhance the learning process in sustainable education ( Delgado-Plaza *et al.*, 2021; Kabadayi *et al.*, 2022). Another study with pre-service teachers also suggested the incorporation of digital storytelling, focusing on online sustainability science courses; digital storytelling introduces the intricate concepts of sustainability science to individuals without a specialized background in the field ( Shelton *et al.*, 2016).

Another case study in Chile offers real-world proof of how ICT can improve community-based Education for Sustainable Development (ESD). It involved creating a theoretical framework that combined learning theories from ICT and ESD, and findings revealed that digital science communication effectively fostered deep and transformative learning, locally rooted ecological literacy, active engagement, and the adoption of sustainable living principles and practices within certain community members ( Aguayo & Eames, 2017).

In the mixed-level education study domain, it was found that digital science communication impacted sustainability literacy. The study using the Eco online platform showed positive results, indicating that the Eco platform effectively enhanced participants’ environmental literacy and systems thinking. Eco was found to strengthen awareness of the ecological impact of human actions on ecosystems. This suggests that Eco could be a valuable tool for improving environmental consciousness related to ecosystems, with recommendations for future use ( Fjællingsdal & Klöckner, 2019). Another study by Rodríguez-Loinaz *et al*. in 2022 demonstrated that innovative ICT tools empowered teachers to engage students in citizen science activities tailored to their local environment.

### 3.4 Obstacles and variables associated with the application of digital science communication

In the primary education domain, the author of the first study recommends that virtual reality (VR) should enhance traditional primary education study methods rather than replace them entirely. This approach helps children develop a systemic worldview, expanding their horizons, even at a young age. However, it has been noted that children may initially focus on using the VR game rather than fully grasping sustainable development concepts. In addition, the price of those VR headsets and WIFI access can also be an obstacle to using them more widely ( Aguayo & Eames, 2023).

In secondary education, it is acknowledged that changing environmental attitudes and behaviors in a single classroom session can be challenging, suggesting extended interventions with active methodologies to foster positive emotions and beliefs. Another study indicates that ICT positively impacts academic progress, with video games more appealing to boys than girls (gender bias). In addition, video games covering some sustainability curricula are lacking, thus often requiring the adaptation of existing video game content to meet specific educational needs ( Martínez-Borreguero *et al.*, 2020).

At the tertiary education level, digital science communication tools like e-learning courses can enhance student-centered, in-depth learning, especially in sustainable design, when combined with hands-on experiences and diverse teaching methods. However, it is essential to note that online tools cannot fully replace in-person instruction. Integrating systems thinking, crucial for teaching sustainability, presents a pedagogical challenge when merging ICT and sustainable development ( Altomonte *et al.*, 2016; Hilty & Huber, 2018).

In the professional training domain, age does not significantly influence technology adoption for virtual teaching, and ESD directly contributes to achieving SDGs ( Delgado-Plaza *et al.*, 2021). Another Study with pre-service teachers found a fascinating point regarding the design of the online course. Participants who raised concerns about accountability noted that they could achieve favorable results in the interactive quizzes even without comprehensively viewing all the assigned content. In addition, students mentioned difficulties such as the inability to rewind or fast-forward within the videos ( Shelton *et al.*, 2016).

Today, we are using more technology but also growing distant from our local natural places, which are crucial for our environment. To address this, the study at the community level aimed to use technology to reconnect people with nature and each other in culturally meaningful ways ( Aguayo & Eames, 2017).

The studies with different academic levels revealed exciting findings. Gender did not show significant differences, but age did. People between 50 and 60 responded differently from those below 30, possibly due to younger individuals being more adapted to technology. This highlights the need for further research to explore the technology gap between age groups and consider factors like economic status, cultural background, or geographic region ( Colás-Bravo & Quintero-Rodríguez, 2023). In the study with the Eco platform, all participants were male despite varying education levels. Future research should aim for greater gender diversity to avoid potential bias in research outcomes ( Fjællingsdal & Klöckner, 2019).

Therefore, the most frequently reported challenges relate to technological accessibility, including the cost of equipment and internet access, as well as pedagogical challenges in integrating digital tools effectively. Other factors, such as age differences and gender-related preferences, were reported less consistently.

### 3.5 Evidence on achieving designated SDGs targets 4.7, 12.8 & 13.3

Mixed reality (MR) immersive learning positively changes children’s behavior in primary education, with increased environmental awareness and eco-friendly practices. Serious game interventions at this level also sparked fresh insights and improved student habits, as noted by both students and teachers. Additionally, serious games help children understand prospective thinking ( Aguayo & Eames, 2023; Veronica & Calvano, 2020).

In secondary education, Information and Communication Technology activities enhanced sustainability literacy. Digital storytelling engaged students and advanced scientific education and environmental literacy in a marine pollution context. Similarly, socio-scientific online argumentation patterns fostered social responsibility and sustainability attitudes while connecting classroom knowledge to real-life situations ( Andriopoulou *et al.*, 2022; Martínez-Borreguero *et al.*, 2020; Tsai, 2018).

At the tertiary level, virtual reality and hands-on courses effectively interested and engaged learners in environmental action. Social media played a pivotal role in supporting sustainability discussions. Technology-enhanced learning in sustainable design boosted motivation and engagement, contributing to students’ attitudes toward sustainability. Connecting content with students’ fields of study facilitated their participation in sustainability discussions ( Altomonte *et al.*, 2016; Gatti *et al.*, 2019; Hilty & Huber, 2018; Istenic Starcic *et al.*, 2018; Liu *et al.*, 2019).

In the professional training domain, online interactive databases provide access to educational content related to sustainability. Training for instructors in food nutrition, energy efficiency, and sustainable agriculture within Education for Sustainable Development (ESD) enhances sustainability literacy. Engagement with online platforms fostered sustainable behaviors and environmentally conscious actions, including innovation and supportiveness. Interactive digital stories proved valuable in engaging future teachers and improving their learning outcomes ( Delgado-Plaza *et al.*, 2021; Horng *et al.*, 2022; Kabadayi *et al.*, 2022; Shelton *et al.*, 2016).

Using Information and Communication Technology (ICT) tools to promote socio-ecological sustainability in community education for sustainable development (ESD) holds significant potential. These technology-enhanced learning systems can help people gain knowledge, improve critical thinking, change their attitudes, and take concrete actions for socio-ecological sustainability ( Aguayo & Eames, 2017).

The study, which included a mixed level of education, such as YouTube’s role in informal learning, highlights its relevance and significance as a tool for education for sustainable development. ( Colás-Bravo & Quintero-Rodríguez, 2023). The study involving the Eco platform found that Eco significantly improved participants’ environmental literacy and systems thinking. This enhanced understanding extends to complex ecosystem dynamics and contemporary environmental issues, such as biodiversity loss and overfishing ( Fjællingsdal & Klöckner, 2019). The study by Rodríguez-Loinaz *et al*. in 2022 supports the idea that educational initiatives can align science education and sustainable development, equipping students with global skills to address 21st-century sustainability challenges.

### 3.6 Word frequency among the selected articles

A word frequency analysis was conducted using NVIVO 12 software to identify the ten most frequently occurring terms within the selected articles, as presented in Table 2. Across all studies, the relevance and proximity of the topics to the target study groups emerged as critical factors strongly influencing the effectiveness of sustainability literacy enhancement through digital science communication.

**Table 2.** Word Frequency in the selected articles.

Word	Count	Weighted Percentage
learning	1644	1,23%
education	1230	0,92%
students	932	0,70%
sustainability	905	0,68%
environmental	818	0,61%
game	795	0,60%
sustainable	675	0,51%
study	543	0,41%
development	534	0,40%
research	516	0,39%

The word cloud visualization highlights key recurring terms such as “learning,” “education,” “students,” “sustainability,” “environmental,” and “game.” This reinforces the central role of digital tools in promoting sustainability literacy across various educational levels and contexts.

## 4. Discussion

This review provides a comprehensive synthesis of how digital science communication contributes to sustainability literacy across different educational contexts, digital tools, and implementation conditions. The findings highlight three key dimensions aligned with the study objectives: (1) the educational settings and geographical distribution of research, (2) the types of digital tools and approaches employed, and (3) their impacts, associated challenges, and contributions to sustainability-related learning outcomes.

Across these dimensions, the results indicate that digital science communication plays a significant role in shaping learners’ understanding, attitudes, and engagement with sustainability, particularly through interactive, experiential, and flexible learning environments. A wide range of digital tools has been applied across educational levels. Extended Reality (XR) technologies, including Mixed Reality (MR), Virtual Reality (VR), and Augmented Reality (AR), have been integrated into educational settings at both primary and tertiary levels to deepen students’ understanding of sustainability ( Aguayo & Eames, 2023; Liu *et al.*, 2019). In addition to XR technologies, various educational interventions such as serious games and simulation games have been employed to influence the sustainable behavior of students. These interactive and experiential learning methods are particularly effective in teaching complex concepts related to sustainability, as they allow students to experiment with different scenarios and see the immediate impact of their decisions at primary and tertiary education levels, demonstrating their versatility and effectiveness ( Fjællingsdal & Klöckner, 2019; Mercer *et al.*, 2017; Robin *et al.*, 2017; Veronica & Calvano, 2020).

E-learning approaches have also played a significant role in modern education, particularly in secondary and tertiary education. Tools such as explanatory videos, WebQuests, and Digital Storytelling (DST) are used extensively through online portals and mobile applications. These methods provide flexible and accessible ways for students to learn at their own pace and convenience, enhancing their understanding of various subjects, including sustainability ( Altomonte *et al.*, 2016; Andriopoulou *et al.*, 2022; Delgado-Plaza *et al.*, 2021; Hilty & Huber, 2018; Horng *et al.*, 2022; Istenic Starcic *et al.*, 2018; Shelton *et al.*, 2016). Furthermore, methods like the Socio-Scientific Issues of Online Argumentation Patterns (SOAP) and social media platforms have been utilized to analyze the impact of educational interventions. These methods help educators understand how students engage with sustainability content and measure the effectiveness of these interventions in fostering positive attitudes towards sustainability ( Colás-Bravo & Quintero-Rodríguez, 2023; Fjællingsdal & Klöckner, 2019; Rodríguez-Loinaz *et al.*, 2022; Tsai, 2018).

Additionally, a case study in Chile examines digital science communication’s role in community-based learning about sustainable development and environmental challenges in southern Chile, revealing that digital science communication effectively fostered profound and transformative education, locally rooted ecological literacy, active engagement, and the adoption of sustainable living principles and practices within certain community members ( Aguayo & Eames, 2017). Lastly, some studies employing mixed sample groups explore informal learning on platforms like YouTube, assess the effectiveness of serious games like Eco in raising awareness, and investigate how citizen science supported by ICT can enhance transformative teaching in education for sustainability.

From a theoretical perspective, these findings can be interpreted through constructivist and transformative learning approaches. Constructivist learning theory suggests that learners actively build knowledge through interaction and experience, which explains the effectiveness of immersive and interactive tools such as XR and serious games ( Schunk, 2020). At the same time, transformative learning highlights the role of critical reflection in reshaping learners’ perspectives and values, which is essential for fostering sustainability-related attitudes and behaviours ( Taylor & Cranton, 2012). These processes are closely linked to behaviour change, as education for sustainability requires not only knowledge acquisition but also shifts in attitudes and everyday practices ( Steg & Vlek, 2009).

Despite these benefits, several challenges limit the effective implementation of digital science communication. Common barriers include limited familiarity with digital tools, design constraints, and issues related to accessibility, such as the cost of equipment and internet infrastructure ( Colás-Bravo & Quintero-Rodríguez, 2023; Fjællingsdal & Klöckner, 2019; Rodríguez-Loinaz *et al.*, 2022). Technically, these challenges can limit opportunities for meaningful interaction and critical reflection, which are central to both constructivist and transformative learning processes. As a result, the potential of digital tools to support behavioural change for the long term may not be fully realiseded.

Overall, the findings suggest that digital science communication enhances sustainability literacy through several interconnected mechanisms. These include:

• Interactive Communication Capability: Both students/learners and professionals or community members benefit from the ability to engage in interactive communication facilitated by digital science communication. This dynamic engagement promotes a more profound understanding and participation in sustainability-related learning and initiatives.
• Contextualized Knowledge Availability: Digital tools deliver context-specific knowledge tailored to diverse communities and environments’ unique needs and challenges. This ensures that sustainability literacy is relevant and applicable to real-world contexts.
• Flexible Learning Opportunities: Digital science communication offers flexible learning opportunities regarding timing and pace. Learners can access educational content conveniently, accommodating individual preferences. This flexibility enhances the accessibility and inclusivity of sustainability education.

From a practical perspective, these findings have important implications for educators and policymakers. Educational institutions should prioritise the integration of interactive and experiential digital tools, such as immersive technologies and game-based learning, when designing sustainability curricula. However, effective implementation requires adequate teacher training, thoughtful instructional design, and alignment with pedagogical objectives. In addition, institutions should adopt a balanced investment strategy that combines high-impact immersive technologies, such as VR, with more scalable and cost-effective tools, including e-learning platforms and digital storytelling. Effective use of these tools also depends on targeted professional development that equips educators with both technical competencies and pedagogical strategies for supporting sustainability learning. At the same time, policymakers should address digital inequality by improving access to technological infrastructure and resources, particularly in underrepresented regions. Ensuring equitable access is essential for scaling the benefits of digital science communication across different socio-economic and geographical contexts.

By providing students with the ability to virtually explore distant locations or engage in experiments that may be logistically challenging in a traditional school setting, digital science communication transcends these barriers. It empowers learners to access transformative educational experiences that were previously inaccessible or impractical. As a take from this scoping review, we can highlight eight points that can be facilitated by digital science communication to enhance sustainability literacy, including (i) enhanced understanding, (ii) awareness and knowledge, (iii) attitude change, (iv) virtual accessibility, (v) experiential learning, (vi) community engagement, (vii) data-driven learning, and (viii) inclusivity and proximity.

Although many studies report positive short-term impacts on knowledge and attitudes, there is limited evidence of long-term behavioural change. This suggests that while digital tools can support initial engagement and awareness, sustained interventions and well-designed learning experiences are needed to translate these outcomes into lasting sustainable practices. Therefore, its effectiveness depends on how well these tools are integrated into pedagogical practices that promote both cognitive understanding and behavioural change. Greater attention to theoretical grounding on sustainability, long-term evaluation, and equitable access will be essential for maximising the impact of digital approaches in education for sustainability.

## 5. Limitations

This scoping review provides a mapping of the literature on digital science communication in Education for Sustainable Development; however, several limitations should be noted. Firstly, while an initial broad search was conducted across Web of Science (WoS) and Scopus, the detailed eligibility assessment, filtering, and full-text screening were performed solely on the WoS dataset. This decision was based on WoS’s enhanced functionality, including granular disciplinary categorization (“Education Educational Research”) and the ability to filter records by specific Sustainable Development Goals (SDGs), which closely aligned with the thematic focus of this review and are not available in Scopus. Nevertheless, this selective approach may have excluded potentially relevant studies uniquely indexed in Scopus or other databases. Furthermore, a portion of the 309 retrieved records could not be accessed or retrieved, and thus were not screened, potentially limiting the comprehensiveness of the review.

Secondly, the literature included in this review is predominantly drawn from studies conducted in developed countries or the Global North, reflecting an evident geographical concentration. This imbalance limits the transferability of insights to educational and sustainability contexts in the Global South, where digital access, educational infrastructures, and socio-cultural dynamics may differ significantly. The limited representation of low-income country perspectives may be explained by the digital divide, differences in research funding, and unequal access to advanced digital tools used in education. In addition, research from the Global South is often underrepresented in indexed databases such as Web of Science, and language barriers may further reduce its visibility in international journals. Addressing this gap represents a key area for future research.

Additionally, although the review adhered to rigorous PRISMA-ScR methodological guidance, the screening, eligibility assessment, and data charting were conducted by a single reviewer, while procedures were applied consistently and transparently; this may introduce subjective bias in the inclusion of studies and thematic synthesis. Future reviews could strengthen reliability by using multiple independent reviewers and consensus mechanisms.

Finally, as a scoping review, the objective was to map and characterize the breadth of existing evidence rather than to assess the methodological quality of interventions. Therefore, while this review offers a productive overview, it does not provide a critical appraisal of study rigor or outcomes, which should be undertaken in subsequent systematic reviews or meta-analyses.

## 6. Conclusions

This scoping review mapped and synthesized the existing literature on digital science communication in Education for Sustainable Development (ESD), with particular attention to educational contexts, digital tools, and their impacts on sustainability literacy.

Regarding RQ1 (educational contexts and settings), the findings show that digital science communication is predominantly implemented in formal education settings, especially at the tertiary level. The majority of studies originate from Europe and other regions of the Global North, indicating a clear geographical concentration in the current evidence base. Research is largely situated within the education sector, while relatively limited attention has been given to applied fields such as tourism, business, and community development. In addition, most included studies were published after 2015, suggesting increased academic attention following the adoption of the Sustainable Development Goals (SDGs).

Regarding RQ2 (digital tools and approaches), a wide range of digital science communication tools was identified, including immersive technologies (VR, AR, and MR), serious games, simulation-based learning, digital storytelling, and broader e-learning platforms. Across studies, immersive and game-based approaches are the most frequently used, particularly for promoting engagement and experiential learning. In contrast, approaches such as citizen science and structured online argumentation remain less frequently applied, indicating emerging but underdeveloped areas within the field.

Regarding RQ3 (impacts, challenges, and outcomes), the evidence indicates that digital science communication generally enhances sustainability literacy by improving learners’ knowledge, awareness, and engagement with sustainability issues. These effects are most evident in interactive and experiential learning environments. However, the literature also highlights important limitations, including limited justification for tool selection in some studies and a lack of longitudinal evidence demonstrating sustained behavioural change. Few studies include follow-up assessments, which constrains understanding of long-term impacts on learners’ attitudes and practices.

Overall, the findings suggest that digital science communication has strong potential to support sustainability literacy through interactive engagement, contextualised learning, and flexible access to educational resources. At the same time, its effectiveness depends on careful pedagogical integration, appropriate instructional design, and attention to contextual constraints. While some studies raise concerns about potential disconnection from direct environmental experience ( Fletcher, 2017), the overall evidence indicates that well-designed digital approaches can enhance rather than replace environmental engagement and awareness.

However, these conclusions should be interpreted in light of the methodological limitations of this review, including the exclusive reliance on Web of Science for full-text screening and the single-reviewer approach used during study selection and data charting. Future research should prioritise longitudinal designs, multi-reviewer methodologies, and greater geographical diversity, particularly by including underrepresented regions, to better understand the long-term and global applicability of digital science communication in sustainability education.

## Ethics and consent

Ethical approval and consent were not required.

## Data availability statement

No data associated with this article.

## Extended data

Zenodo. Extended Data of Digital Science Communication for Sustainability Literacy: A Scoping Review Paper https://doi.org/10.5281/zenodo.16632684 ( Aung, 2025).

The project contains the following extended data:

• Extended Data Table. Analysis Matrix and NVIVO12 data of reviewed articles

Data are available under the terms of the Creative Commons Attribution 4.0 International license (CC-BY 4.0).

## AI Use disclosure statement

In accordance with the F1000 AI Policy, ChatGPT (GPT-4.5) was used solely to assist with language editing and improving clarity and academic tone. It was also used for general suggestions on structure and wording. No AI tools were used for data analysis, interpretation, or generation of original scientific content. All intellectual contributions remain the responsibility of the authors.

## Reporting guidelines

Zenodo. Extended Data of Digital Science Communication for Sustainability Literacy: A Scoping Review Paper https://doi.org/10.5281/zenodo.16632684 ( Aung, 2025).

The project contains the following reporting guidelines files:

• PRISMA-ScR Checklist
• PRISMA-ScR Flowchart

Data are available under the terms of the Creative Commons Attribution 4.0 International license (CC-BY 4.0).
