# Supplementary material for: Digital science communication for sustainability literacy: A scoping review
Source: Open Res Eur. 2026 Jun 24;5:239. Originally published 2025 Aug 8. [Version 2] doi: 10.12688/openreseurope.20966.2 (PMC13309778; doi:10.12688/openreseurope.20966.2)
Supplement: Peer review history

# Reviewer report: Reviewer response for version 2

**DOI:** 10.21956/openreseurope.25850.r76449

**Published:** 2026-06-27

**Authors:** Subhankar Das

**Affiliations:** 1 Duy Tan University, Da Nang, Vietnam

I am happy with the revisions. No comments.

Are sufficient details of the methods and analysis provided to allow replication by others?

No

Are the rationale for, and objectives of, the Systematic Review clearly stated?

Partly

Is the statistical analysis and its interpretation appropriate?

No

Are the conclusions drawn adequately supported by the results presented in the review?

No

Reviewer Expertise:

Marketing, PLS-SEM, FsQCA

I confirm that I have read this submission and believe that I have an appropriate level of expertise to confirm that it is of an acceptable scientific standard.

# Reviewer report: Reviewer response for version 2

**DOI:** 10.21956/openreseurope.25850.r76450

**Published:** 2026-06-26

**Authors:** Rubén Rivas-de-Roca

**Affiliations:** 1 University of Santiago de Compostela, Santiago, Spain

The authors have made a great effort to address all the reviewer’s concerns. Specifically, the rationale and scientific soundness of the paper have been addressed, answering research questions and offering a proper discussion. Besides that, theoretical sections are better organization.

 There are also enough methodological clarifications of the literature review provided (adherence to the PRISMA-ScR framework). Hence, I do think it is indexable in its current version.

Are sufficient details of the methods and analysis provided to allow replication by others?

Yes

Are the rationale for, and objectives of, the Systematic Review clearly stated?

Partly

Is the statistical analysis and its interpretation appropriate?

Yes

Are the conclusions drawn adequately supported by the results presented in the review?

Yes

Reviewer Expertise:

Communication

I confirm that I have read this submission and believe that I have an appropriate level of expertise to confirm that it is of an acceptable scientific standard.

# Reviewer report: Reviewer response for version 1

**DOI:** 10.21956/openreseurope.22683.r60679

**Published:** 2025-10-01

**Authors:** Subhankar Das

**Affiliations:** 1 Duy Tan University, Da Nang, Vietnam

**Literature Review Requires Expansion**

 The theoretical foundation would benefit from deeper engagement with established frameworks in educational technology adoption, behaviour change theories, and science communication models. Foundational work in these domains appears underrepresented, particularly scholarship predating 2015. This narrow temporal focus risks overlooking significant theoretical developments that inform current practice.

**Methodology Needs Enhancement**

 While the PRISMA-ScR framework provides structure, critical methodological details require elaboration. The rationale for excluding Scopus records after initial screening is mentioned but deserves fuller justification. Why specifically did Web of Science's SDG filtering capabilities outweigh the potential insights from Scopus-indexed studies?

 The single-reviewer screening process, acknowledged as a limitation, should be discussed more transparently in terms of how bias was mitigated. The NVIVO 12 coding process needs operational definitions for each category to ensure reproducibility.

**Sampling and Analysis Need Elaboration**

 The geographical and educational-level distribution of studies deserves quantitative presentation beyond narrative description. Why do European contexts dominate?

 What explains the absence of low-income country perspectives? The thematic synthesis would benefit from inter-coder reliability measures and explicit discussion of how themes were derived, refined, and validated.

**Implications need Deeper Discussion.**

 The review identifies that digital tools enhance sustainability literacy through interactive engagement, contextualised learning, and flexible access. Yet, it insufficiently explores what these findings mean for educational policy, curriculum design, and resource allocation.

 The practical implications for educators implementing these technologies remain underdeveloped. How should institutions prioritise investments in VR versus serious games? What training do educators need? How can digital tools be scaled equitably across different socioeconomic contexts? These questions deserve thorough exploration.

**Conclusions Must Address Objectives Systematically**

 The conclusion should explicitly revisit each stated objective, demonstrating how findings address research questions. The disconnect between ambitious objectives and relatively modest conclusions suggests either overreaching initial aims or insufficient synthesis of findings. Stronger integration between empirical findings and stated goals would substantially improve the review's coherence and impact.

Are sufficient details of the methods and analysis provided to allow replication by others?

No

Are the rationale for, and objectives of, the Systematic Review clearly stated?

Partly

Is the statistical analysis and its interpretation appropriate?

No

Are the conclusions drawn adequately supported by the results presented in the review?

No

Reviewer Expertise:

Marketing, PLS-SEM, FsQCA

I confirm that I have read this submission and believe that I have an appropriate level of expertise to confirm that it is of an acceptable scientific standard, however I have significant reservations, as outlined above.

# Reviewer report: Reviewer response for version 1

**DOI:** 10.21956/openreseurope.22683.r60676

**Published:** 2025-09-25

**Authors:** Maria Alzira Pimenta Dinis

**Affiliations:** 1 University Fernando Pessoa (UFP), Porto, Portugal

There is some relevant similarity and also AI use which is perfectly evident in the language used here and not adequate to a scientific article in my perspective.

 “People everywhere should understand how to live sustainably to solve these challenges and achieve the United Nations Sustainable Development Goals (SDGs). This understanding is called sustainability literacy,”

 The language used compromises the entire manuscript.

 I am unsure if this specific journal requires a structured abstract, which is however unclear in terms of specific findings and also conclusions because this is mainly a remark,

 “Conclusion However, challenges remain, including limited evidence of long-term behavioral impacts and the need for more tailored digital content. Ultimately, this review suggests that digital communication can help reconnect people with nature and deepen their understanding of sustainable practices, while highlighting the need for further research to address persistent gaps.”

 We are in 2025 already, and most references in the introductory section would require updating. In several cases, there is relevant similarity here.

 Please remember that the manuscript will have to answer to the objectives at the end of the introductory section,

 “The primary objectives of this review are: ▪ To identify which digital tools have been applied to pro- mote sustainability literacy and how they are being used; ▪ To assess the educational levels, fields, and locations in which these approaches have been implemented; ▪ To explore the impacts and obstacles of using digital science communication in sustainability education. ▪ To highlight knowledge gaps and inform future empirical studies and policy interventions.”

 These are not exactly parameters. And this caption represents full similarity seen in other studies,

 “Figure 1. Parameters defined in the study.”

 Because we are at the beginning. It would be important to highlight at the end of the caption which sources have authors based on to build this figure.

 If to be clear about inclusion criteria there are parameters missing here. For example the language or if the abstract or the entire text contained this or that.

 “Figure 2. Inclusion criteria.”

 And again for similarity here, in any case, I suggest the author is to revise most lists,

 “2.5. Summary of findings from selected studies using qualitative techniques A qualitative analysis was performed based on the informa- tion in the analysis matrix. Five categories related to the guide questions were coded using NVIVO 12 software. One category was associated with each question.”

 There is in my perspective, no need to refer to other parts of the manuscript here.

 “The outcomes of this investigation are situated within the context of the five categories presented as follows (3.1 to 3.5).”

 We should never include the term findings or results, inside a captions table. As it will imply that those are presented as they are, and already discussed or analysed in that same table, which is obviously not the intention.

 “Table 1. Findings from selected studies, categories, and references.”

 The results needs to be presented in a comprehensive way, that is, is less separated paragraphs and interconnected content

 “Table 1. Findings from selected studies, categories, and references.”

 Figure captions should be as enlightening as possible. So terms as different countries without further contextualization or the time frame should be addressed every time, also we need specific legends to the axes

 “Figure 4. Selected Digital Science Communications Studies Conducted in Different Countries and Corresponding Levels.”

 Please clarify all headings. Application of what given, if evident to the authors,

 “3.4 Obstacles and variables associated with the application”

 Please use upper letter in SDGs...

 “3.5 Evidence on achieving designated sdgs targets 4.7, 12.8 & 13.3”

 And again please verify all captions also see that we cannot have commas but dots within numbers and no need to add percentage next to each value if indicated above

 “Table 2. Word Frequency in the selected articles.”

 Please make sure to articulate the mention to the research questions in relation to the objectives mentioned at the end of the introductory section, there is obviously no need to indicate what SDGS are again or to start the discussion with “methods”,

 “4. Discussion This study conducted a scoping review to examine articles related to the role of digital science communication in enhanc- ing sustainability literacy. It sought to answer five key research questions, including the academic settings and fields where these studies were conducted, the digital communication tools and approaches employed to shape learners’ sustain- ability practices, the impacts of such communication tools on sustainability literacy, the obstacles and variables associated with its application, and the evidence supporting its contribution to achieving Sustainable Development Goals (SDGs) targets 4.7, 12.8 and 13.3 related to sustainability literacy.”

 Avoid redundant commentaries

 “In summary, enhancing”

 Similar statements can be placed, were presented under the form of a box, for example, avoiding the listing,

 “As evident from the scoping literature review, we can highlight eight points that can be facilitated by digital science communication to enhance sustainability literacy. • Enhanced Understanding • Awareness and Knowledge • Attitude Change • Virtual Accessibility • Experiential Learning • Community Engagement • Data-Driven Learning • Inclusivity and Proximity”

 I suggest ending the discussion section with a clear paragraph that highlights the most relevant information deriving from it,

 In my perspective, limitations are to be addressed at the very end of the conclusions section before further prospects

 In addition, the conclusions section should start with a clear context Before methods clear findings, implications, limitations, and further prospects

 Avoid terminology as many studies because we will not know exactly what this means.

 It seems to me that the manuscript is interesting. It could be further worked in terms of streamlining and better translating, what is more relevant in terms of clarifying information. To me a problem relies in the language used that resembles AI in several cases, as the style, which is identifiable as such. Then because this is a review, I believe authors would need to further clarify the relevance and practical implications of this review compared to existing others.

Are sufficient details of the methods and analysis provided to allow replication by others?

Yes

Are the rationale for, and objectives of, the Systematic Review clearly stated?

Yes

Is the statistical analysis and its interpretation appropriate?

Partly

Are the conclusions drawn adequately supported by the results presented in the review?

Partly

Reviewer Expertise:

Sustainability

I confirm that I have read this submission and believe that I have an appropriate level of expertise to confirm that it is of an acceptable scientific standard, however I have significant reservations, as outlined above.

# Reviewer report: Reviewer response for version 1

**DOI:** 10.21956/openreseurope.22683.r59464

**Published:** 2025-09-15

**Authors:** Rubén Rivas-de-Roca

**Affiliations:** 1 University of Santiago de Compostela, Santiago, Spain

1. The text deals with a very relevant and timely topic, analyzing how digital science communication contributes to SDGs. The authors develop a scientific scoping review of prior scholarship based on PRISMA framework. Although the sample is small, the method allows to learn more about how digital science communication enhances learners’ sustainability literacy between 2000 and 2023. This literature review is strong, but it could be reinforced with some foundational contributions.

 2. The manuscript employs a clear methodology (search strategy through Boolean operators), together with objectives. However, there are too many research questions (five). It would be better to group them and answer each one separately in the conclusions.

 3. It is identified how the selected digital science communications studies were conducted in different countries, but there is no information on the authors’ countries of origin. This is key to understanding the dynamics of scientific production.

 4. Moreover, I really appreciate the evidence provided on achieving designated SDGs targets by the sample. Nevertheless, the weight of each trend on digital communication tools is not present. The same goes for the section on obstacles and variables associated with the application.

 5. The authors apply a narrative study that indicates which was the first study or the differences between educational levels. This type of approach offers some insightful findings, but it might be complemented by figures or tables that illustrate the value of each variable, even if the sample is only 20 articles (as is generally done in Table 1).

 6. In my opinion, the discussion section works really well as the results are put in relation to prior scholarship. Besides that, the articles spotlight eight countries that can be facilitated by digital science communication to ameliorate sustainability literacy. This is a clear contribution of the current study.

 Summing up, the manuscript is an interesting contribution in terms of sustainability literacy, but three main changes: expanding a bit the theoretical background, responding to research questions that may be reworked, and strengthen the analysis of some results.

Are sufficient details of the methods and analysis provided to allow replication by others?

Yes

Are the rationale for, and objectives of, the Systematic Review clearly stated?

Partly

Is the statistical analysis and its interpretation appropriate?

Yes

Are the conclusions drawn adequately supported by the results presented in the review?

Yes

Reviewer Expertise:

Communication

I confirm that I have read this submission and believe that I have an appropriate level of expertise to confirm that it is of an acceptable scientific standard, however I have significant reservations, as outlined above.
